# Examining the upper frequency limit of dynamic cerebral autoregulation: Considerations across the cardiac cycle during eucapnia

**DOI:** 10.1113/EP091719

**Published:** 2024-10-09

**Authors:** Joel S. Burma, Matthew G. Neill, Elizabeth K. S. Fletcher, Brooke E. Dennett, Nathan E. Johnson, Raelyn Javra, James K. Griffiths, Jonathan D. Smirl

**Affiliations:** ^1^ Cerebrovascular Concussion Laboratory, Faculty of Kinesiology University of Calgary Calgary Alberta Canada; ^2^ Sport Injury Prevention Research Centre, Faculty of Kinesiology University of Calgary Calgary Alberta Canada; ^3^ Human Performance Laboratory, Faculty of Kinesiology University of Calgary Calgary Alberta Canada; ^4^ Libin Cardiovascular Institute of Alberta University of Calgary Calgary Alberta Canada; ^5^ Alberta Children's Hospital Research Institute University of Calgary Calgary Alberta Canada; ^6^ Hotchkiss Brain Institute University of Calgary Calgary Alberta Canada; ^7^ Integrated Concussion Research Program University of Calgary Calgary Alberta Canada; ^8^ Biomedical Engineering University of Calgary Calgary Alberta Canada

**Keywords:** cerebral blood flow, cerebral blood velocity, cerebral pressure–flow relationship, dynamic cerebral autoregulation upper limit, squat–stand manoeuvre, transfer function analysis

## Abstract

There are differences within the literature regarding the upper frequency cut‐off point of the dynamic cerebral autoregulation (CA) high‐pass filter. The projection pursuit regression approach has demonstrated that the upper frequency limit is ∼0.07 Hz, whereas another approach [transfer function analysis (TFA) phase approaching zero] indicated a theoretical upper frequency limit for the high‐pass filter of 0.24 Hz. We investigated how these limits accurately represent the CA upper frequency limit, in addition to extending earlier findings with respect to biological sexes and across the cardiac cycle. Sixteen participants (nine females and seven males) performed repeated squat–stand manoeuvres at frequencies of 0.05, 0.10, 0.15, 0.20 and 0.25 Hz, with insonation of the middle and posterior cerebral arteries. Linear regression modelling with adjustment for sex and order of squat completion was used to compared TFA gain and phase with 0.25 Hz (above the theoretical limit of CA). The upper frequency limit of CA with TFA gain was within the range of 0.05–0.10 Hz, whereas TFA phase was within the range of 0.20–0.25 Hz, and consistent between vessels, between sexes and across the cardiac cycle. Females displayed greater middle cerebral artery gain compared with males (all *P <* 0.047), and no phase differences were present (all *P >* 0.072). Although sex‐specific differences were present for specific TFA metrics at a given frequency, the upper frequency limit of autoregulation was similar between cerebral conduit vessels, cardiac cycle phase and biological sex. Future work is warranted to determine whether an upper frequency limit exists with respect to hysteresis analyses.

## Abstract (highlights)

- **What is the central question of this study?**
  There is conflict in the current literature regarding the upper frequency limit of dynamic cerebral autoregulation, with proposed limits ranging from 0.07 to 0.24 Hz. The vast majority of previous work on the upper cerebral autoregulation frequency limit has been performed in males and with mean blood pressure and cerebral blood velocity.
- **What is the main finding and its importance?**
  The upper frequency limit for gain was 0.05–0.10 Hz and for phase it was 0.20–0.25 Hz. This occurred irrespective of cardiac cycle phase or biological sex.

## 1 INTRODUCTION

Cerebral autoregulation (CA) is a fundamental regulatory mechanism within the cerebrovasculature and refers to the ability of the cerebral blood vessels to dampen changes in blood pressure (Brassard et al., 2021; Panerai et al., 2023). Originally, it was proposed CA functioned to maintain cerebral blood flow constant at ∼50 mL/min/100 g brain tissue across a wide range of perfusion pressures (50–150 mmHg) (Lassen, 1959; Paulson et al., 1990). However, more recent research investigations and review papers have called into question this ‘CA Dogma’ (Brassard et al., 2021; Willie et al., 2014). It is now accepted there is a very narrow (or absent) plateau region, with cerebral blood flow changing more for a given decrease in blood pressure than increase (Labrecque et al., 2022; Numan et al., 2014). Furthermore, the original approaches to assess CA were obtained through measurements over several minutes, which was coined ‘static CA’ (Lassen, 1959), whereas transcranial Doppler ultrasound enables measurements of cerebral blood velocity (CBv; surrogate for cerebral blood flow; Skow et al., 2022) in real time, which has opened the field of ‘dynamic CA’ (Aaslid et al., 1989, 1982; Tiecks et al., 1995).

Dynamic CA relates to the ability of the cerebrovasculature to dampen alterations in CBv for a transient change in blood pressure (Aaslid et al., 1989; Birch et al., 1995; Brassard et al., 2023; Zhang et al., 1998). There have been numerous methods proposed for assessing dynamic CA, which have included the rate of regulation (Aaslid et al., 1989), autoregulatory index (Panerai et al., 2003; Tiecks et al., 1995), projection pursuit regression (PPR; Hamner & Tan, 2014; Tan, 2012; Tan et al., 2013) and, most commonly, transfer function analysis (TFA; Claassen et al., 2016; Panerai et al., 2023; Zhang et al., 1998). The principal notion underlying these dynamic CA assessment techniques is that the dampening of blood pressure oscillations within the cerebrovasculature functions as a high‐pass filter that allows slower changes in blood pressure to be dampened effectively, whereas faster changes in blood pressure are passed through to the brain (Zhang et al., 1998). In the seminal paper by Zhang et al. (1998), this was suggested to occur around a frequency of ∼0.20 Hz (Zhang et al., 1998).

The first study to investigate the upper frequency limit of CA was performed with spontaneous blood pressure oscillations and alterations in arterial carbon dioxide levels and found that the upper frequency limit ranged from 0.094 (hypercapnia resulting from 8% carbon dioxide inhalation) to 0.167 Hz (hypocapnia at −10 mmHg from normocapnia), with an upper frequency limit for eucapnia at 0.122 Hz (Panerai et al., 2019). The upper frequency limit of the high‐pass filter was determined by Panerai et al. (2019) as the frequency at which the TFA phase reached and oscillated around zero. It has also been well established that the use of spontaneous blood pressure oscillations (or even oscillatory lower‐body negative pressure‐induced oscillations) when processing dynamic CA data via TFA leads to high levels of variability and poor reproducibility, which confounds the interpretability of these data sets (Smirl et al., 2015). Other research groups have attempted to delineate the upper frequency limit from PPR analyses via oscillatory lower‐body negative pressure (Hamner & Tan, 2014; Tan et al., 2013; Taylor et al., 2014). Through this analytical approach, it was revealed that oscillations occurring at >0.07 Hz were largely linear in nature because the driven oscillations in blood pressure were largely being passed directly onto CBv metrics (Taylor et al., 2014). Based upon these divergent findings, there appear to be discrepancies for the upper frequency limit of CA depending on which approach is adopted (TFA phase or PPR). Finally, it should be noted that the aforementioned studies into the upper frequency limit of autoregulation examined the mean middle cerebral artery (MCA) independently. It is thus unknown which frequency best identifies the upper limit for CA and the extent that this might differ based on the cardiac cycle component and vessel of interest (Burma, Copeland, Macaulay, et al., 2020; Smirl et al., 2018; Wright et al., 2018).

It has been proposed in the most recent white paper from the International Cerebrovascular Research Network (CARNet) that the optimal method for enhancing the signal‐to‐noise ratio when using TFA to assess dynamic CA is repeated squat–stand manoeuvres (SSMs; Panerai et al., 2023). As highlighted by the authors of the TFA assessment, it is possible the use of repeated SSMs could reveal a different upper frequency limit cut‐off point, and an investigation using this technique ‘would be of considerable interest’ (Panerai et al., 2019). Therefore, in the present investigation we built upon these prior studies examining the upper frequency limit of the dynamic CA and sought to determine which frequency (0.07 Hz from PPR or ∼0.20 Hz with TFA phase) encapsulates the upper limit of the high‐pass CA filter. As recommended by Panerai et al. (2019), a series of repeated SSMs were performed in eucapnic conditions ranging from 0.05 to 0.25 Hz within each phase of the cardiac cycle (diastole, mean and systole). Consistent with previous work, it was hypothesized that there will be decreases in TFA phase from the slower oscillations at 0.05 Hz until they reach and maintain values around zero, which should occur at ∼0.20 Hz (Zhang et al., 1998). It was hypothesized that the TFA gain metric will have a comparable upper frequency limit to the TFA phase metric of ∼0.20 Hz. Furthermore, previous work has demonstrated systole to be more effective at buffering oscillations in blood pressure, compared to mean or diastole (Burma, Copeland, Wright, et al., 2020; Smirl et al., 2018; Wright et al., 2018). It was also hypothesized that the TFA phase data associated with diastole will reach zero at a lower frequency (0.15 Hz) and that the systolic TFA phase data will reach zero at a higher frequency (0.25 Hz).

## 2 MATERIALS AND METHODS

### 2.1 Ethical approval

The study received ethical approval by the Calgary Conjoint Health Research Ethics Board (REB20‐1662). Except for registration in a database, all study protocols were completed in accordance with the guidelines put forth within the *Declaration of Helsinki* (revised version 2008, excluding the registration of the study). Before study commencement, all protocols were thoroughly explained, instrumentation was explained, all questions were answered, and written consent was obtained. Data for this study were collected in November 2023.

### 2.2 Participants and study design

A convenience sample of 17 participants were recruited from the university community (nine females and eight males). Data from one male participant were excluded owing to poor signal quality, hence the final sample included a total of 16 participants (nine females and seven males). Females were an average age of 25.0 ± 3.2 years of age, with a body mass index of 24.5 ± 2.1 kg/m^2^, and males were an average age of 27.9 ± 3.5 years of age, with a body mass index of 26.1 ± 5.5 kg/m^2^. Testing of females was not controlled for with respect to their menstrual cycle phase because prior research has shown that there is minimal influence of the menstrual cycle on CA metrics (Favre & Serrador, 2019; Johnson et al., 2023). No participants reported a history of any cerebrovascular, neurological, musculoskeletal and/or respiratory diseases. Participants were instructed to refrain from exercise for a minimum of 6 h before data collection (Burma, Copeland, Macaulay, et al., 2020, Burma, Copeland, Wright, et al., 2020) and to abstain from alcohol, caffeine, smoking and vaping for ≥8 h (Ainslie et al., 2008, 2007; Smirl, Lucas, Lewis, et al., 2015, 2014).

To minimize the likelihood of external confounding factors influencing the outcome metrics, a randomized crossover design was used, with all individuals completing all five repeated SSM frequencies within a single laboratory visit. This augments the internal validity for a given study design, because participants act as their own control (Mills et al., 2009).

### 2.3 Instrumentation

A transcranial Doppler ultrasound (Doppler Box, DWL USA, San Juan Capistrano, CA, USA) was used to measure CBv through the transtemporal window on each side of the cranium for the MCA and posterior cerebral artery (PCA; Purkayastha & Sorond, 2012; Willie et al., 2011). Once the MCA and PCA were identified and confirmed through visual and carotid compression checks, the probes were locked in place with a fitted headframe (DWL USA). Heart rate based upon R–R intervals was measured through a three‐lead ECG, using a second lead approach (FE 231, ADInstruments, Colorado Springs CO, USA) (Atwood & Wadlund, 2015). Beat‐to‐beat blood pressure was measured via finger photoplethysmography on the left middle finger, with a brachial cuff to correct systemic blood pressure values to the level of the heart (Finapres NOVA, Finapres Medical Systems, Amsterdam, The Netherlands). This method has previously been shown to assess reliably the alterations in beat‐to‐beat blood pressure, which are well correlated with intra‐arterial recordings and can be used during dynamic CA assessments (Omboni et al., 1993; Ortega‐Gutierrez et al., 2014; Sammons et al., 2007). Finally, a mouthpiece and inline gas analyser (ML 206, ADInstruments) were used to measure the end‐tidal partial pressure of carbon dioxide ($P_{\mathrm{ET},CO_{2}}$), which were calibrated before participant arrival with a known gas concentration (5.00% carbon dioxide, 15.94% oxygen and balance nitrogen) and room air (0.03% carbon dioxide, 20.93% oxygen and 78.09% nitrogen). During all SSMs, $P_{\mathrm{ET},CO_{2}}$ values were monitored continuously to ensure that they were as comparable as possible between all tasks, because carbon dioxide has been demonstrated to be an important confounding factor to control for when deriving autoregulatory estimates (Aaslid et al., 1989; Birch et al., 1995; Brassard et al., 2023; Yoshida et al., 2018)

### 2.4 Experimental protocol

All data collection occurred during a single laboratory visit to the Cerebrovascular Concussion Laboratory at the University of Calgary. These sessions occurred during the normal working day of an individual, which has been shown to have minimal dynamic CA alterations associated with diurnal variation (Burma, Copeland, Macaulay, et al., 2020). The laboratory conditions were tightly controlled across all sessions for the following environmental factors: barometric pressure, 662.0 ± 1.8 mmHg; humidity, 17.5% ± 4.0%; and temperature, 22.0°C ± 0.5°C. The elevation of the laboratory was 1111 m above sea level.

**FIGURE 1:**
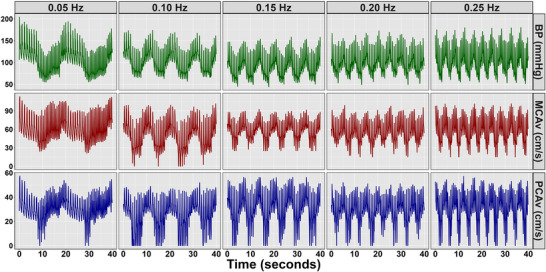
Representative BP, MCAv and PCAv waveforms from one participant during all five squat–stand manoeuvre frequencies of interest. Abbreviations: BP, blood pressure; MCAv, middle cerebral artery velocity; PCAv, posterior cerebral artery velocity.

Once the equipment was attached and locked in place, participants completed five randomized sets of SSMs at set frequencies of 0.05, 0.10, 0.15, 0.20 and 0.25 Hz (Burma, Roy, Kennedy, et al., 2024). Representative blood pressure and CBv data can be seen in Figure 1. A frequency of 0.05 Hz was chosen because it falls within the very low‐frequency range of autoregulation (0.02–0.07 Hz) (Zhang et al., 1998), which provides an assessment of the metabolic, endothelial, neurogenic and/or myogenic influences on dynamic CA. The frequency of 0.10 Hz was chosen because it falls within the low‐frequency range (0.07–0.20 Hz) (Zhang et al., 1998), accounting for the influence of sympathetic innervation on dynamic CA (Hamner & Tan, 2014; Hamner et al., 2010). This frequency also encompasses the upper frequency limit of dynamic CA established through the hypercapnic trials (0.094 ± 0.040 Hz) established through spontaneous TFA (Panerai et al., 2019). The frequency of 0.15 Hz was included because it encompasses the upper frequency limit of dynamic CA established through the hypocapnic trials (0.167 ± 0.036 Hz) established through spontaneous TFA (Panerai et al., 2019). The frequency of 0.20 Hz was included because it was the previously proposed upper frequency limit of dynamic CA within the first CARNet white paper (Claassen et al., 2016). Finally, the frequency of 0.25 Hz was included because this was above the extreme upper frequency limit of dynamic CA (0.24 Hz) calculated as a Gaussian distribution from the data presented by Panerai et al. (2019). In order to ensure that the participants completed the repeated SSMs at the frequency of interest, the manoeuvres were performed in rhythm with a metronome at 6 (0.05 Hz), 12 (0.10 Hz), 18 (0.15 Hz), 24 (0.20 Hz) and 30 beats/min (0.25 Hz).

### 2.5 Data processing

Blood pressure and CBv values were calculated across the cardiac cycle (Burma, Copeland, Macaulay, et al., 2020; Newel et al., 2022; Panerai et al., 2023; Smirl et al., 2018; Wright et al., 2018). Systolic and diastolic values were obtained as the maximum and minimum values from each pulsatile waveform, respectively. Mean values were calculated as the average of all data points calculated across each waveform. Data were sampled at 1000 Hz using commercially available software (PowerLab 16/30 ML880, ADInstruments). Researchers visually inspected all traces and manually corrected all artefacts by interpolating the correct systolic peaks in all traces as required (<0.1% of all traces). The R–R interval was used to calculate heart rate. Calculations of $P_{\mathrm{ET},CO_{2}}$ were done using breath‐to‐breath peak values of the partial pressure of carbon dioxide. All data processing was in accordance with the standards put forward by the CARNet white papers (Claassen et al., 2016; Panerai et al., 2023). LabChart (ADInstruments) was used to quantify physiological outputs from the raw data during the SSMs (e.g., mean arterial blood pressure, CBv and $P_{\mathrm{ET},CO_{2}}$), whereas commercially available software (Ensemble‐R v.1.0.42, R&D Canvas, Wellington, New Zealand) was used to derive the CA power spectrum density and TFA estimates.

### 2.6 Transfer function analysis

A Welch smoothing method was used to improve spectral estimates, whereby beat‐to‐beat blood pressure and CBv waveforms were spline interpolated and resampled at 4 Hz (Claassen et al., 2016; Panerai et al., 2023). As previously described, it is recommended that a 300 s recording is used to derive TFA estimates from SSMs (Burmaa, Miutz, Newel, et al., 2021). Data from the 300 s recording were detrended and passed through a Hanning window (five windows, 50% overlap, 100 s per window). As previously described (Burma, Copeland, Macaulay, et al., 2021; Wright et al., 2018), cross‐spectrums were taken between blood pressure and CBv at each phase of the cardiac cycle (e.g., diastolic arterial blood pressure and diastolic CBv). For each cardiac cycle component, the values were then divided by the respective autospectrum. Autoregulatory TFA estimates were then computed at the driven point estimates of 0.05, 0.10, 0.15, 0.20 and 0.25 Hz. This was completed for the diastolic, mean and systolic aspects of the cardiac cycle at all frequency point estimates to produce TFA estimates within each component (i.e., diastolic blood pressure to diastolic CBv) (Burma, Copeland, Wright, et al., 2020; Panerai et al., 2023; Smirl et al., 2018; Wright et al., 2018).

The outcome measures of interest included TFA coherence, phase, gain and normalized gain (nGain). Coherence describes the fraction of the input power (blood pressure) that is linearly expressed by the output power (CBv) on an arbitrary scale ranging from zero (no linear relationship) to one (complete linear relationship) (Zhang et al., 1998). Phase describes the temporal alignment (radians), whereas absolute gain (in centimetres per second per millimetre of mercury) quantifies the amplitude modulation within the cerebral pressure–flow relationship (Zhang et al., 1998). Normalized gain (expressed as a percentage per millimetre of mercury) was reported in addition to absolute gain, because it has shown to be more reliable (Burma, Copeland, Macaulay, et al., 2020; Smirl et al., 2015). Given the TFA computation method used (i.e., spectral smoothing, 50% overlap and five windows), the critical value for coherence was determined in alignment with the CARNet white paper at an α value of 0.01 (Panerai et al., 2023). Thus, the a priori coherence threshold was set at 0.46. Phase wraparound was not present in any of the data samples.

### 2.7 Sample size calculation

An a priori sample size calculation was determined using G*Power (v.3.1.9.6) with a fixed model linear multiple regression. The meta‐analyses pooled estimates and 95% confidence intervals (95% CI) presented by Burma and colleagues (2024), accumulating all previous studies using SSMs to derive phase and gain measures, were used to calculate the required sample size: 0.05 Hz phase (0.74; 95% CI: 0.66–0.82), 0.10 Hz phase (0.48; 95% CI: 0.40–0.56), 0.05 Hz gain (0.67; 95% CI: 0.58–0.65) and 0.10 Hz gain (0.87; 95% CI: 0.82–0.92). It has been proposed that a phase of zero and a gain of one correspond to the absence of buffering and hence CA (Zhang et al., 1998). Using this supposition, the aforementioned phase and gain metrics produced a large Cohen's *d* effect size (>1.50) compared with a reference value of zero or one, which produced a Cohen's *f* effect size of 0.75. This was reduced to 0.50 to ensure that the study was adequately powered at frequencies of >0.15 Hz. Using a Cohen's *f* effect size of 0.50, α of 0.05, power of 0.80, one tail and three predictors (frequency of SSMs, sex and order of SSMs completed), a sample of 15 was required. A one‐tailed directional test was used because gain is known to increase with a greater frequency, while phase decreases to a value of zero (Zhang et al., 1998).

### 2.8 Statistical analysis

All statistical analysis were completed within R‐Studio (Posit, PBC;, v.2022.7.1.554). Biological sex differences in absolute cerebrovascular, cardiovascular and respiratory parameters were compared using Mann–Whitney U‐tests. Harmonic regression analyses were completed on the blood pressure and CBv waveforms to determine the consistency of the oscillatory waveforms at 0.05, 0.10, 0.15, 0.20 and 0.25 Hz. This technique allows for the decomposition of a time‐series signal into its constituent sinusoidal components, each characterized by a specific frequency, amplitude and phase. From this, the stability of the amplitude and phase of the oscillatory signal over the 5 min was quantified and computed via coefficient of variation (CoV). Linear regressions were also completed for all TFA outcome metrics (i.e., coherence, phase, absolute gain and nGain), with frequency [0.25 Hz (reference), 0.05, 0.10, 0.15 and 0.20 Hz] being the predictor variable and with adjustment for sex [female (reference) or male] and order of frequency of SSMs completed. Although the SSMs were randomized, the inclusion of this factor controlled for the order of SSMs in the statistical models. The linear regressions were completed for each metric within each phase of the cardiac cycle (e.g., diastole MCA gain, mean MCA gain) (Panerai et al., 2023; Smirl et al., 2018). The frequency of 0.25 Hz was chosen as the reference group based on past work denoting that this is well above the upper frequency limit for all variables (Panerai et al., 2019). Therefore, if regulation is present, one would expect this to be greater than the value at 0.25 Hz. This will identify the frequency when a metric achieves a plateau (as seen in gain) and/or when the phase values are no longer greater than zero. Data are displayed as the mean ± 95% CI, to approximate the estimated population mean. Additionally, the 95% CI for the phase values help to identify when this is no longer different from a value of zero, which would be contained within the 95% CIs. Finally, to understand whether the coherence value influenced the TFA phase, gain and nGain estimates, these were binned into coherence ranges of 0.70–0.75, 0.75–0.80, 0.80–0.85, 0.85–0.90, 0.90–0.95 and >0.95. The value of α was set a priori at 0.05.

## 3 RESULTS

**FIGURE 2:**
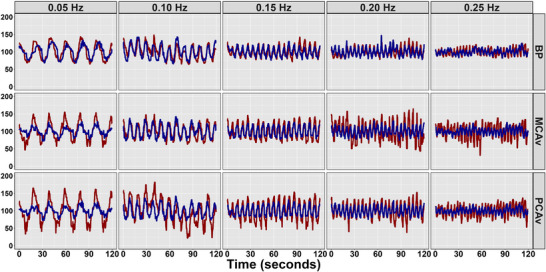
Representative traces of BP, MCAv and PCAv for the most (red) and least (blue) variable participants across five frequencies (0.05, 0.10, 0.15, 0.20 and 0.25 Hz) of repeated squat–stand manoeuvres. Abbreviations: BP, blood pressure; MCAv, middle cerebral artery velocity; PCAv, posterior cerebral artery velocity.

Absolute cerebrovascular, cardiovascular and respiratory parameters at baseline and during all SSMs are displayed in Table 1, stratified by sex. Table 2 displays the values for all TFA estimates during all squat–stand frequencies stratified by sex. These values are displayed graphically in supplemental figures ([Link], [Link], [Link], [Link]). Table 3 displays the linear regression output estimates and 95% Cis, with the associated *P*‐values. The harmonic regression analyses produced a phase CoV of <1% in blood pressure, middle cerebral artery velocity (MCAv) and posterior cerebral artery velocity (PCAv) oscillations, while the amplitude CoV was 14.8%, 18.0% and 19.4% for blood pressure, MCAv and PCAv, respectively. Greater CoV amplitudes were noted generally to decrease with an increasing amplitude: 0.05 Hz (19.9%), 0.10 Hz (22.0%), 0.15 Hz (17.3%), 0.20 Hz (14.7%) and 0.25 Hz (13.2%). Data from the two participants with the least and most variation are displayed in Figure 2, normalized to the average value from both individuals to allow for a direct comparison.

**TABLE 1:** Absolute cerebrovascular, cardiovascular and respiratory parameters during all squat–stand manoeuvres in 16 participants (9 females and 7 males).

Variable	Sex	Baseline	0.05 Hz	0.10 Hz	0.15 Hz	0.20 Hz	0.25 Hz
Diastole MCAv (cm/s)	Combined	45.3 (39.7, 50.9)	38.1 (32.1, 44.2)	36.5 (29.9, 43.1)	38.1 (30.5, 45.6)	37.4 (30.1, 44.7)	39.8 (32.5, 47.1)
	Female	**52.1 (47.0, 57.2)**	**46.3 (40.5, 52.0)**	**45.1 (39.2, 51.1)**	**45.8 (35.5, 56.1)**	**44.0 (33.3, 54.7)**	**46.9 (36.0, 57.7)**
	Male	**36.5 (29.3, 43.7)**	**27.7 (23.9, 31.5)**	**25.4 (19.1, 31.8)**	**28.1 (21.0, 35.2)**	**28.9 (21.9, 35.9)**	**30.7 (25.6, 35.7)**
Mean MCAv (cm/s)	Combined	61.4 (53.5, 69.2)	58.6 (51.4, 65.8)	58.5 (51.0, 66.1)	59.6 (50.1, 69.1)	58.4 (49.6, 67.2)	61.4 (52.3, 70.5)
	Female	**71.2 (63.6, 78.9)**	**68.1 (60.5, 75.8)**	**68.4 (61.3, 75.4)**	**69.8 (56.8, 82.8)**	**67.7 (55.7, 79.7)**	**71.6 (59.4, 83.7)**
	Male	**48.7 (40.6, 56.7)**	**46.4 (42.3, 50.4)**	**45.9 (38.8, 52.9)**	**46.5 (39.4, 53.6)**	**46.4 (39.1, 53.7)**	**48.4 (42.5, 54.2)**
Systolic MCAv (cm/s)	Combined	94.4 (82.4, 106.3)	100.4 (90.0, 110.7)	102.3 (91.3, 113.3)	103.4 (88.7, 118.1)	101.3 (88.8, 113.9)	105.8 (92.7, 119.0)
	Female	**109.0 (96.7, 121.3)**	**111.3 (97.7, 124.9)**	**114.5 (102.3, 126.7)**	**117.4 (96.2, 138.5)**	**114.4 (97.0, 131.7)**	**120.0 (102.5, 137.4)**
	Male	**75.6 (63.1, 88.1)**	**86.3 (76.7, 95.9)**	**86.6 (73.1, 100.0)**	**85.4 (71.5, 99.3)**	**84.6 (74.5, 94.6)**	**87.7 (76.5, 98.9)**
Diastolic PCAv (cm/s)	Combined	29.8 (24.8, 34.7)	21.9 (17.4, 26.3)	19.4 (14.1, 24.8)	22.1 (16.6, 27.6)	23.0 (18.3, 27.8)	23.5 (18.0, 29.1)
	Female	29.4 (22.5, 36.2)	**26.0 (19.4, 32.7)**	**23.2 (14.9, 31.4)**	**25.6 (17.2, 34.0)**	**25.9 (18.8, 33.1)**	**26.9 (18.0, 35.9)**
	Male	30.3 (20.7, 40.0)	**16.5 (12.8, 20.3)**	**14.6 (7.96, 21.2)**	**17.6 (10.1, 25.1)**	**19.3 (12.5, 26.1)**	**19.2 (12.5, 25.9)**
Mean PCAv (cm/s)	Combined	40.9 (34.8, 47.0)	35.4 (29.6, 41.2)	34.7 (27.8, 41.7)	38.7 (32.8, 44.6)	37.5 (31.7, 43.3)	39.5 (33.7, 45.3)
	Female	40.9 (32.4, 49.4)	38.7 (29.0, 48.5)	36.4 (24.4, 48.5)	**42.7 (33.7, 51.6)**	39.6 (29.6, 49.6)	**43.3 (34.3, 52.4)**
	Male	40.9 (29.1, 52.7)	31.1 (25.2, 37.1)	32.5 (23.8, 41.3)	**33.6 (25.7, 41.4)**	34.9 (28.0, 41.8)	**34.5 (27.3, 41.7)**
Systolic PCAv (cm/s)	Combined	63.0 (53.4, 72.7)	63.2 (54.7, 71.7)	64.9 (56.3, 73.6)	67.3 (57.5, 77.0)	66.8 (58.7, 74.9)	67.8 (58.3, 77.4)
	Female	61.2 (47.2, 75.3)	66.5 (53.6, 79.4)	65.7 (53.4, 77.9)	71.0 (56.0, 86.1)	68.8 (56.3, 81.3)	71.2 (56.2, 86.2)
	Male	65.4 (47.5, 83.2)	59.0 (45.3, 72.7)	64.0 (47.4, 80.6)	62.4 (47.1, 77.8)	64.2 (50.7, 77.7)	63.5 (48.7, 78.3)
Diastolic BP (mmHg)	Combined	71.1 (61.9, 80.2)	60.9 (52.8, 69.1)	58.7 (50.0, 67.4)	60.1 (51.3, 69.0)	63.2 (53.9, 72.6)	61.8 (55.1, 68.5)
	Female	76.0 (61.3, 90.8)	67.6 (55.9, 79.3)	65.2 (52.8, 77.6)	63.9 (50.6, 77.1)	69.3 (54.8, 83.7)	65.3 (56.3, 74.3)
	Male	64.7 (52.9, 76.5)	52.3 (42.0, 62.6)	50.4 (38.1, 62.7)	55.3 (41.0, 69.6)	55.5 (43.5, 67.5)	57.2 (45.2, 69.2)
Mean BP (mmHg)	Combined	89.5 (79.6, 99.4)	82.1 (73.3, 90.9)	82.1 (72.7, 91.6)	84.1 (74.6, 93.5)	87.5 (77.3, 97.6)	86.3 (79.1, 93.5)
	Female	94.7 (78.9, 110.4)	89.3 (76.0, 102.7)	89.2 (75.1, 103.3)	89.4 (75.2, 103.5)	93.6 (76.8, 110.4)	90.3 (79.7, 100.9)
	Male	82.8 (69.5, 96.2)	72.9 (63.3, 82.4)	72.9 (61.1, 84.8)	77.3 (63.1, 91.4)	79.6 (68.8, 90.3)	81.1 (70.0, 92.2)
Systolic BP (mmHg)	Combined	135.3 (123.0, 147.6)	135.4 (125.3, 145.4)	138.4 (126.5, 150.3)	142.9 (131.2, 154.5)	144.3 (131.0, 157.6)	143.7 (133.6, 153.8)
	Female	140.1 (120.8, 159.5)	140.7 (123.6, 157.8)	143.6 (122.9, 164.2)	147.0 (128.6, 165.5)	149.0 (124.7, 173.4)	144.6 (127.2, 162.1)
	Male	129.1 (110.2, 147.9)	128.5 (117.8, 139.1)	131.8 (118.8, 144.7)	137.6 (119.9, 155.2)	138.2 (127.5, 148.9)	142.5 (129.0, 155.9)
Heart rate (beats/min)	Combined	84.3 (78.0, 90.5)	97.8 (87.8, 107.7)	95.9 (87.7, 104.1)	99.0 (91.8, 106.1)	102.9 (95.3, 110.5)	106.4 (98.0, 114.9)
	Female	83.3 (73.7, 93.0)	96.0 (84.6, 107.5)	93.6 (82.8, 104.5)	99.7 (89.4, 110.0)	102.5 (90.9, 114.1)	105.0 (93.3, 116.8)
	Male	85.5 (74.9, 96.1)	100.0 (78.1, 121.9)	98.8 (82.5, 115.0)	98.1 (84.7, 111.4)	103.4 (90.2, 116.6)	108.3 (91.9, 124.7)
$P_{\mathrm{ET},CO_{2}}$ (mmHg)	Combined	38.6 (36.8, 40.4)	38.0 (36.3, 39.7)	38.9 (37.0, 40.8)	38.2 (35.7, 40.7)	38.8 (37.0, 40.7)	39.0 (37.1, 40.9)
	Female	38.0 (34.8, 41.3)	37.4 (34.4, 40.3)	38.2 (34.8, 41.6)	38.4 (34.8, 42.0)	37.8 (34.6, 41.0)	38.4 (34.8, 41.9)
	Male	39.3 (37.9, 40.8)	38.8 (36.9, 40.7)	39.8 (38.2, 41.5)	38.1 (33.4, 42.8)	40.2 (38.5, 41.8)	39.7 (38.6, 40.8)
Respiratory rate (breaths/min)	Combined	16.2 (14.2, 18.1)	19.3 (17.6, 20.9)	19.5 (17.5, 21.6)	20.6 (18.4, 22.8)	20.7 (18.5, 22.8)	20.9 (18.3, 23.5)
	Female	16.2 (13.3, 19.0)	18.8 (16.6, 21.0)	19.5 (16.7, 22.2)	20.5 (17.0, 24.0)	21.4 (18.2, 24.5)	20.7 (17.0, 24.5)
	Male	16.2 (12.6, 19.8)	19.9 (16.6, 23.1)	19.6 (15.6, 23.6)	20.7 (17.0, 24.3)	19.8 (16.1, 23.4)	21.0 (16.1, 25.9)

*Note*: Data are displayed as the mean (95% confidence intervals). Bold denotes significant biological sex difference. Sex differences in physiological parameters were assessed using the Mann–Whitney *U*‐test.

Abbreviations: BP, blood pressure; MCAv, middle cerebral artery velocity; PCAv, posterior cerebral artery velocity; $P_{\mathrm{ET},CO_{2}}$, end‐tidal partial pressure of carbon dioxide.

**TABLE 2:** Transfer functional analysis estimates produced during the five squat–stand manoeuvre frequencies in 16 participants (9 females and 7 males).

Variable	Cardiac	Component	Sex	0.05 Hz	0.10 Hz	0.15 Hz	0.20 Hz	0.25 Hz
Coherence	Diastole	MCAv	Female	0.99 (0.98, 1.00)	0.99 (0.97, 1.01)	0.99 (0.96, 1.00)	0.97 (0.93, 1.00)	0.99 (0.98, 1.00)
			Male	0.99 (0.97, 1.00)	0.99 (0.98, 1.00)	0.99 (0.99, 1.00)	0.98 (0.94, 1.00)	0.98 (0.96, 1.00)
			Combined	0.99 (0.98, 0.99)	0.99 (0.98, 1.00)	0.99 (0.97, 1.00)	0.97 (0.95, 1.00)	0.99 (0.98, 0.99)
		PCAv	Female	0.99 (0.98, 1.00)	0.99 (0.97, 1.00)	0.98 (0.95, 1.00)	0.99 (0.97, 1.00)	0.99 (0.98, 1.00)
			Male	0.99 (0.98, 1.00)	0.99 (0.99, 1.00)	0.99 (0.99, 1.00)	0.97 (0.94, 1.00)	0.98 (0.96, 1.00)
			Combined	0.99 (0.98, 1.00)	0.99 (0.98, 1.00)	0.99 (0.97, 1.00)	0.98 (0.97, 1.00)	0.98 (0.97, 0.99)
	Mean	MCAv	Female	0.97 (0.95, 1.00)	0.99 (0.99, 1.00)	0.99 (0.97, 1.00)	0.98 (0.95, 1.00)	0.99 (0.98, 1.00)
			Male	0.98 (0.96, 1.00)	0.99 (0.99, 1.00)	0.99 (0.99, 1.00)	0.98 (0.96, 1.00)	0.98 (0.96, 1.00)
			Combined	0.98 (0.96, 1.00)	0.99 (0.99, 1.00)	0.99 (0.98, 1.00)	0.98 (0.96, 1.00)	0.99 (0.98, 1.00)
		PCAv	Female	0.99 (0.98, 1.00)	0.99 (0.98, 1.00)	0.99 (0.97, 1.00)	0.99 (0.98, 1.00)	0.99 (0.98, 1.00)
			Male	0.98 (0.96, 1.00)	0.99 (0.99, 1.00)	0.99 (0.99, 1.00)	0.98 (0.96, 1.00)	0.98 (0.95, 1.00)
			Combined	0.99 (0.97, 1.00)	0.99 (0.99, 1.00)	0.99 (0.98, 1.00)	0.99 (0.98, 1.00)	0.99 (0.97, 1.00)
	Systole	MCAv	Female	0.95 (0.92, 0.98)	0.98 (0.97, 0.99)	0.95 (0.90, 0.99)	0.91 (0.84, 0.98)	0.86 (0.79, 0.93)
			Male	0.97 (0.96, 0.99)	0.99 (0.98, 0.99)	0.93 (0.87, 0.98)	0.93 (0.91, 0.95)	0.84 (0.76, 0.93)
			Combined	0.96 (0.94, 0.98)	0.98 (0.98, 0.99)	0.94 (0.91, 0.97)	0.92 (0.88, 0.96)	0.85 (0.81, 0.90)
		PCAv	Female	0.95 (0.89, 1.00)	0.97 (0.93, 1.00)	0.96 (0.91, 1.00)	0.92 (0.85, 1.00)	0.87 (0.78, 0.95)
			Male	0.94 (0.87, 1.00)	0.96 (0.90, 1.00)	0.94 (0.90, 0.99)	0.88 (0.78, 0.98)	0.82 (0.72, 0.92)
			Combined	0.94 (0.91, 0.98)	0.96 (0.93, 0.99)	0.95 (0.92, 0.98)	0.90 (0.85, 0.96)	0.85 (0.79, 0.90)
Phase	Diastole	MCAv	Female	0.66 (0.58, 0.74)	0.40 (0.33, 0.47)	0.25 (0.19, 0.30)	0.18 (0.08, 0.27)	0.05 (−0.09, 0.20)
			Male	0.65 (0.37, 0.94)	0.37 (0.29, 0.46)	0.29 (0.22, 0.37)	0.26 (0.15, 0.37)	0.06 (−0.07, 0.19)
			Combined	0.66 (0.55, 0.77)	0.39 (0.34, 0.44)	0.27 (0.23, 0.31)	0.21 (0.14, 0.28)	0.06 (−0.03, 0.14)
		PCAv	Female	0.62 (0.50, 0.74)	0.34 (0.25, 0.43)	0.23 (0.16, 0.31)	0.16 (0.08, 0.25)	0.03 (−0.14, 0.19)
			Male	0.53 (0.40, 0.66)	0.40 (0.35, 0.44)	0.32 (0.23, 0.41)	0.27 (0.12, 0.42)	0.05 (−0.05, 0.15)
			Combined	0.58 (0.50, 0.66)	0.37 (0.32, 0.41)	0.27 (0.22, 0.33)	0.21 (0.13, 0.29)	0.04 (−0.05, 0.13)
	Mean	MCAv	Female	0.73 (0.62, 0.83)	0.48 (0.32, 0.65)	0.21 (0.13, 0.29)	0.12 (0.03, 0.21)	0.00 (−0.12, 0.13)
			Male	0.61 (0.46, 0.76)	0.39 (0.34, 0.44)	0.27 (0.21, 0.33)	0.23 (0.12, 0.34)	0.05 (−0.07, 0.17)
			Combined	0.68 (0.59, 0.76)	0.44 (0.35, 0.53)	0.24 (0.19, 0.29)	0.17 (0.10, 0.24)	0.02 (−0.06, 0.10)
		PCAv	Female	0.71 (0.55, 0.86)	0.38 (0.27, 0.49)	0.22 (0.13, 0.32)	0.12 (0.04, 0.20)	−0.01 (−0.14, 0.12)
			Male	0.56 (0.42, 0.69)	0.39 (0.36, 0.42)	0.27 (0.18, 0.35)	0.22 (0.07, 0.38)	−0.01 (−0.14, 0.11)
			Combined	0.64 (0.54, 0.74)	0.38 (0.33, 0.43)	0.24 (0.19, 0.30)	0.17 (0.09, 0.25)	−0.01 (−0.09, 0.07)
	Systole	MCAv	Female	1.06 (0.81, 1.31)	0.72 (0.52, 0.93)	0.49 (0.32, 0.66)	0.26 (0.05, 0.47)	−0.00 (−0.22, 0.22)
			Male	0.80 (0.47, 1.14)	0.51 (0.36, 0.65)	0.55 (0.20, 0.89)	0.29 (−0.05, 0.63)	−0.21 (−0.46, 0.04)
			Combined	0.96 (0.77, 1.14)	0.64 (0.50, 0.77)	0.51 (0.37, 0.65)	0.27 (0.11, 0.43)	−0.08 (−0.24, 0.07)
		PCAv	Female	0.96 (0.62, 1.30)	0.71 (0.42, 1.01)	0.48 (0.25, 0.71)	0.27 (0.07, 0.47)	0.08 (−0.11, 0.26)
			Male	0.76 (0.44, 1.08)	0.47 (0.19, 0.75)	0.50 (0.18, 0.82)	0.26 (0.04, 0.48)	−0.01 (−0.39, 0.37)
			Combined	0.88 (0.66, 1.09)	0.61 (0.42, 0.80)	0.49 (0.33, 0.65)	0.27 (0.14, 0.40)	0.04 (−0.12, 0.20)
Gain	Diastole	MCAv	Female	0.95 (0.80, 1.10)	1.14 (0.93, 1.35)	1.16 (1.01, 1.30)	1.29 (0.99, 1.58)	1.39 (1.10, 1.69)
			Male	0.93 (0.59, 1.27)	1.19 (0.92, 1.47)	1.07 (0.90, 1.24)	1.08 (0.79, 1.37)	1.01 (0.61, 1.42)
			Combined	0.94 (0.79, 1.09)	1.16 (1.02, 1.31)	1.12 (1.02, 1.21)	1.19 (1.00, 1.38)	1.23 (0.99, 1.46)
		PCAv	Female	0.74 (0.57, 0.90)	0.93 (0.78, 1.08)	1.06 (0.90, 1.21)	1.01 (0.89, 1.13)	0.97 (0.80, 1.13)
			Male	0.74 (0.47, 1.01)	1.00 (0.72, 1.29)	0.99 (0.70, 1.28)	0.94 (0.68, 1.20)	0.90 (0.50, 1.31)
			Combined	0.74 (0.61, 0.87)	0.96 (0.83, 1.10)	1.02 (0.89, 1.16)	0.98 (0.86, 1.10)	0.94 (0.76, 1.12)
	Mean	MCAv	Female	0.76 (0.62, 0.89)	1.08 (0.89, 1.26)	1.22 (0.98, 1.46)	1.19 (0.96, 1.42)	1.25 (1.02, 1.49)
			Male	0.70 (0.53, 0.87)	0.87 (0.62, 1.13)	0.91 (0.65, 1.16)	0.87 (0.63, 1.12)	0.83 (0.67, 1.00)
			Combined	0.73 (0.64, 0.82)	0.99 (0.85, 1.13)	1.08 (0.91, 1.26)	1.05 (0.88, 1.22)	1.07 (0.89, 1.25)
		PCAv	Female	0.51 (0.39, 0.63)	0.71 (0.57, 0.85)	0.81 (0.64, 0.98)	0.79 (0.64, 0.93)	0.81 (0.63, 0.98)
			Male	0.53 (0.41, 0.64)	0.70 (0.50, 0.91)	0.71 (0.53, 0.89)	0.72 (0.58, 0.85)	0.68 (0.52, 0.84)
			Combined	0.52 (0.44, 0.59)	0.71 (0.60, 0.81)	0.76 (0.65, 0.87)	0.75 (0.67, 0.84)	0.75 (0.64, 0.86)
	Systole	MCAv	Female	0.41 (0.31, 0.51)	0.64 (0.48, 0.79)	0.75 (0.58, 0.91)	0.89 (0.71, 1.08)	0.79 (0.58, 1.00)
			Male	0.37 (0.26, 0.49)	0.50 (0.29, 0.70)	0.54 (0.19, 0.88)	0.54 (0.30, 0.77)	0.76 (0.32, 1.20)
			Combined	0.40 (0.33, 0.46)	0.58 (0.46, 0.70)	0.66 (0.51, 0.82)	0.75 (0.59, 0.91)	0.78 (0.60, 0.96)
		PCAv	Female	0.31 (0.18, 0.45)	0.40 (0.25, 0.55)	0.45 (0.28, 0.62)	0.48 (0.26, 0.71)	0.40 (0.24, 0.55)
			Male	0.24 (0.17, 0.31)	0.37 (0.14, 0.61)	0.45 (0.03, 0.87)	0.45 (0.11, 0.78)	0.71 (0.23, 1.19)
			Combined	0.28 (0.20, 0.36)	0.39 (0.28, 0.50)	0.45 (0.28, 0.62)	0.47 (0.30, 0.63)	0.53 (0.33, 0.73)
nGain	Diastole	MCAv	Female	2.09 (1.79, 2.39)	2.56 (2.15, 2.97)	3.18 (2.45, 3.91)	2.98 (2.54, 3.42)	3.06 (2.50, 3.62)
			Male	3.46 (1.93, 4.99)	4.66 (3.11, 6.22)	4.18 (2.77, 5.58)	3.46 (2.41, 4.51)	3.34 (1.41, 5.27)
			Combined	2.69 (2.00, 3.37)	3.48 (2.65, 4.32)	3.62 (2.93, 4.30)	3.19 (2.73, 3.65)	3.18 (2.42, 3.94)
		PCAv	Female	2.97 (1.98, 3.96)	4.35 (2.76, 5.94)	4.24 (2.84, 5.65)	4.16 (2.72, 5.60)	3.56 (2.71, 4.42)
			Male	4.47 (2.61, 6.34)	6.43 (4.84, 8.03)	5.57 (3.01, 8.13)	4.63 (2.98, 6.28)	4.59 (2.10, 7.09)
			Combined	3.67 (2.71, 4.64)	5.32 (4.18, 6.46)	4.86 (3.60, 6.12)	4.38 (3.44, 5.32)	4.04 (2.95, 5.14)
	Mean	MCAv	Female	1.13 (0.94, 1.32)	1.57 (1.41, 1.73)	1.75 (1.53, 1.97)	1.76 (1.59, 1.93)	1.77 (1.53, 2.02)
			Male	1.52 (1.24, 1.79)	1.89 (1.52, 2.27)	1.92 (1.63, 2.21)	1.75 (1.44, 2.07)	1.72 (1.46, 1.98)
			Combined	1.30 (1.12, 1.47)	1.71 (1.53, 1.89)	1.82 (1.66, 1.98)	1.76 (1.61, 1.90)	1.75 (1.59, 1.91)
		PCAv	Female	1.26 (0.93, 1.60)	1.80 (1.53, 2.07)	1.86 (1.55, 2.17)	1.85 (1.68, 2.02)	1.80 (1.65, 1.95)
			Male	1.73 (1.49, 1.96)	2.21 (1.81, 2.61)	2.18 (1.68, 2.67)	1.95 (1.57, 2.33)	2.00 (1.59, 2.41)
			Combined	1.48 (1.25, 1.70)	1.99 (1.76, 2.23)	2.01 (1.75, 2.27)	1.90 (1.73, 2.07)	1.89 (1.71, 2.08)
	Systole	MCAv	Female	0.39 (0.27, 0.51)	0.57 (0.43, 0.71)	0.67 (0.53, 0.81)	0.82 (0.68, 0.95)	0.71 (0.50, 0.93)
			Male	0.44 (0.32, 0.56)	0.56 (0.36, 0.75)	0.61 (0.26, 0.96)	0.59 (0.38, 0.79)	1.02 (0.26, 1.78)
			Combined	0.41 (0.34, 0.49)	0.57 (0.47, 0.66)	0.65 (0.51, 0.78)	0.73 (0.61, 0.84)	0.83 (0.55, 1.12)
		PCAv	Female	0.55 (0.18, 0.91)	0.61 (0.40, 0.82)	0.63 (0.42, 0.84)	0.68 (0.44, 0.92)	0.57 (0.38, 0.76)
			Male	0.41 (0.27, 0.55)	0.59 (0.23, 0.96)	0.74 (0.02, 1.45)	0.69 (0.13, 1.25)	1.23 (0.25, 2.22)
			Combined	0.49 (0.29, 0.68)	0.60 (0.44, 0.76)	0.68 (0.41, 0.95)	0.68 (0.46, 0.91)	0.85 (0.45, 1.26)

*Note*: Data are displayed as the mean (95% confidence intervals).

Abbreviations: MCAv, middle cerebral artery velocity; nGain, normalized gain; PCAv, posterior cerebral artery velocity.

**TABLE 3:** Linear regression output for all transfer function analysis variables in 16 participants (9 females and 7 males).

Variable	Cardiac	Component	0.05 Hz	0.10 Hz	0.15 Hz	0.20 Hz	Sex	SSMs order
Coherence	Diastole	MCAv	−0.01 (−0.03, 0.01); *P* = 0.313	0.01 (−0.01, 0.02); *P* = 0.423	0.00 (−0.01, 0.02); *P* = 0.569	−0.00 (−0.02, 0.01); *P* = 0.616	0.00 (−0.01, 0.01); *P* = 0.780	0.00 (−0.00, 0.00); *P* = 0.899
		PCAv	0.01 (−0.01, 0.02); *P* = 0.354	0.01 (−0.01, 0.02); *P* = 0.342	0.01 (−0.01, 0.02); *P* = 0.487	−0.00 (−0.02, 0.01); *P* = 0.768	−0.00 (−0.01, 0.01); *P* = 0.543	0.00 (−0.00, 0.00); *P* = 0.512
	Mean	MCAv	0.00 (−0.02, 0.02); *P* = 0.905	0.01 (−0.01, 0.03); *P* = 0.563	0.00 (−0.02, 0.02); *P* = 0.709	−0.01 (−0.03, 0.01); *P* = 0.359	0.00 (−0.01, 0.01); *P* = 0.918	−0.00 (−0.00, 0.00); *P* = 0.859
		PCAv	−0.00 (−0.01, 0.01); *P* = 0.949	0.01 (−0.01, 0.02); *P* = 0.314	0.01 (−0.01, 0.02); *P* = 0.404	0.00 (−0.01, 0.01); *P* = 0.930	−0.00 (−0.01, 0.01); *P* = 0.480	0.00 (−0.00, 0.00); *P* = 0.227
	Systole	MCAv	**0.10 (0.06, 0.15); *P* < 0.001**	**0.13 (0.08, 0.17); *P* < 0.001**	**0.08 (0.04, 0.13); *P* < 0.001**	**0.06 (0.02, 0.11); *P* = 0.004**	−0.00 (−0.03, 0.03); *P* = 0.896	0.00 (−0.01, 0.01); *P* = 0.835
		PCAv	**0.09 (0.03, 0.15); *P* = 0.002**	**0.11 (0.05, 0.17); *P* < 0.001**	**0.10 (0.04, 0.15); *P* = 0.001**	0.04 (−0.02, 0.09); *P* = 0.218	−0.02 (−0.06, 0.01); *P* = 0.195	0.01 (−0.01, 0.02); *P* = 0.253
Phase	Diastole	MCAv	**0.68 (0.58, 0.78); *P* < 0.001**	**0.44 (0.34, 0.54); *P* < 0.001**	**0.24 (0.14, 0.34); *P* < 0.001**	**0.17 (0.07, 0.27); *P* = 0.001**	0.00 (−0.06, 0.07); *P* = 0.909	0.01 (−0.01, 0.04); *P* = 0.234
		PCAv	**0.57 (0.47, 0.66); *P* < 0.001**	**0.36 (0.26, 0.45); *P* < 0.001**	**0.26 (0.17, 0.35); *P* < 0.001**	**0.20 (0.11, 0.29); *P* < 0.001**	0.04 (−0.02, 0.10); *P* = 0.170	0.02 (−0.00, 0.04); *P* = 0.120
	Mean	MCAv	**0.62 (0.52, 0.72); *P* < 0.001**	**0.35 (0.25, 0.45); *P* < 0.001**	**0.23 (0.13, 0.33); *P* < 0.001**	**0.18 (0.08, 0.28); *P* < 0.001**	0.02 (−0.04, 0.09); *P* = 0.454	0.01 (−0.01, 0.03); *P* = 0.410
		PCAv	**0.68 (0.58, 0.77); *P* < 0.001**	**0.42 (0.32, 0.52); *P* < 0.001**	**0.28 (0.18, 0.37); *P* < 0.001**	**0.20 (0.10, 0.29); *P* < 0.001**	0.00 (−0.06, 0.06); *P* = 0.898	0.02 (−0.01, 0.05); *P* = 0.176
	Systole	MCAv	**1.07 (0.86, 1.28); *P* < 0.001**	**0.75 (0.53, 0.96); *P* < 0.001**	**0.62 (0.41, 0.83); *P* < 0.001**	**0.41 (0.21, 0.62); *P* < 0.001**	−0.12 (−0.25, 0.01); *P* = 0.073	−0.00 (−0.05, 0.05); *P* = 0.970
		PCAv	**0.87 (0.64, 1.10); *P* < 0.001**	**0.61 (0.38, 0.84); *P* < 0.001**	**0.49 (0.26, 0.71); *P* < 0.001**	**0.27 (0.05, 0.49); *P* = 0.019**	−0.10 (−0.24, 0.04); *P* = 0.173	0.01 (−0.04, 0.06); *P* = 0.626
Gain	Diastole	MCAv	**−0.34 (−0.52, −0.15); *P* = 0.001**	−0.08 (−0.27, 0.11); *P* = 0.410	0.01 (−0.17, 0.20); *P* = 0.881	0.01 (−0.17, 0.20); *P* = 0.910	**−0.26 (−0.38, −0.15); *P* < 0.001**	−0.02 (−0.06, 0.03); *P* = 0.442
		PCAv	**−0.20 (−0.39, −0.00); *P* = 0.045**	0.03 (−0.17, 0.22); *P* = 0.796	0.09 (−0.11, 0.28); *P* = 0.375	0.05 (−0.14, 0.24); *P* = 0.574	−0.03 (−0.15, 0.09); *P* = 0.630	−0.00 (−0.04, 0.04); *P* = 0.994
	Mean	MCAv	**−0.29 (−0.52, −0.07); *P* = 0.012**	−0.07 (−0.29, 0.16); *P* = 0.556	−0.11 (−0.34, 0.11); *P* = 0.318	0.00 (−0.22, 0.23); *P* = 0.990	**−0.14 (−0.28, −0.00); *P* = 0.046**	−0.03 (−0.08, 0.02); *P* = 0.305
		PCAv	**−0.24 (−0.37, −0.11); *P* < 0.001**	−0.05 (−0.18, 0.08); *P* = 0.425	0.00 (−0.13, 0.14); *P* = 0.952	−0.01 (−0.14, 0.12); *P* = 0.845	−0.06 (−0.14, 0.02); *P* = 0.157	−0.00 (−0.03, 0.03); *P* = 0.843
	Systole	MCAv	**−0.37 (−0.55, −0.18); *P* < 0.001**	−0.18 (−0.37, 0.00); *P* = 0.054	−0.10 (−0.29, 0.09); *P* = 0.285	0.02 (−0.16, 0.21); *P* = 0.790	**−0.16 (−0.28, −0.04); *P* = 0.009**	−0.01 (−0.05, 0.03); *P* = 0.577
		PCAv	**−0.27 (−0.47, −0.06); *P* = 0.011**	−0.16 (−0.36, 0.05); *P* = 0.125	−0.10 (−0.30, 0.11); *P* = 0.344	−0.09 (−0.29, 0.11); *P* = 0.369	0.03 (−0.09, 0.16); *P* = 0.607	−0.00 (−0.05, 0.04); *P* = 0.889
nGain	Diastole	MCAv	**−0.44 (−0.66, −0.23); *P* < 0.001**	−0.04 (−0.25, 0.18); *P* = 0.738	0.08 (−0.13, 0.29); *P* = 0.460	−0.00 (−0.21, 0.21); *P* = 0.991	**0.16 (0.03, 0.29); *P* = 0.019**	0.03 (−0.02, 0.08); *P* = 0.254
		PCAv	−0.20 (−1.62, 1.21); *P* = 0.777	**1.45 (0.03, 2.86); *P* = 0.045**	0.96 (−0.45, 2.37); *P* = 0.181	0.61 (−0.78, 2.00); *P* = 0.385	**1.24 (0.36, 2.12); *P* = 0.006**	0.17 (−0.15, 0.48); *P* = 0.297
	Mean	MCAv	−0.47 (−1.33, 0.40); *P* = 0.283	0.31 (−0.55, 1.18); *P* = 0.472	0.46 (−0.41, 1.32); *P* = 0.296	−0.01 (−0.86, 0.85); *P* = 0.986	**1.03 (0.49, 1.57); *P* < 0.001**	0.10 (−0.09, 0.30); *P* = 0.289
		PCAv	**−0.40 (−0.67, −0.13); *P* = 0.004**	0.12 (−0.15, 0.38); *P* = 0.392	0.12 (−0.14, 0.39); *P* = 0.361	−0.00 (−0.27, 0.26); *P* = 0.983	**0.30 (0.13, 0.46); *P* = 0.001**	0.04 (−0.02, 0.10); *P* = 0.192
	Systole	MCAv	**−0.42 (−0.64, −0.20); *P* < 0.001**	**−0.27 (−0.48, −0.05); *P* = 0.017**	−0.18 (−0.40, 0.03); *P* = 0.096	−0.10 (−0.31, 0.12); *P* = 0.376	0.01 (−0.13, 0.14); *P* = 0.915	0.00 (−0.05, 0.05); *P* = 0.964
		PCAv	**−0.39 (−0.74, −0.03); *P* = 0.033**	−0.27 (−0.63, 0.08); *P* = 0.130	−0.20 (−0.55, 0.16); *P* = 0.270	−0.20 (−0.55, 0.15); *P* = 0.259	0.12 (−0.10, 0.34); *P* = 0.278	−0.00 (−0.08, 0.08); *P* = 0.951

*Note*: Data are displayed as the mean (95% confidence intervals) with *P*‐values. Predictor variables included frequency (0.25 Hz as reference), sex (female as reference) and the order of SSMs completed. Bold denotes significance.

Abbreviations: MCAv, middle cerebral artery velocity; nGain, normalized gain; PCAv, posterior cerebral artery velocity; SSMs, squat–stand manoeuvres.

**FIGURE 3:**
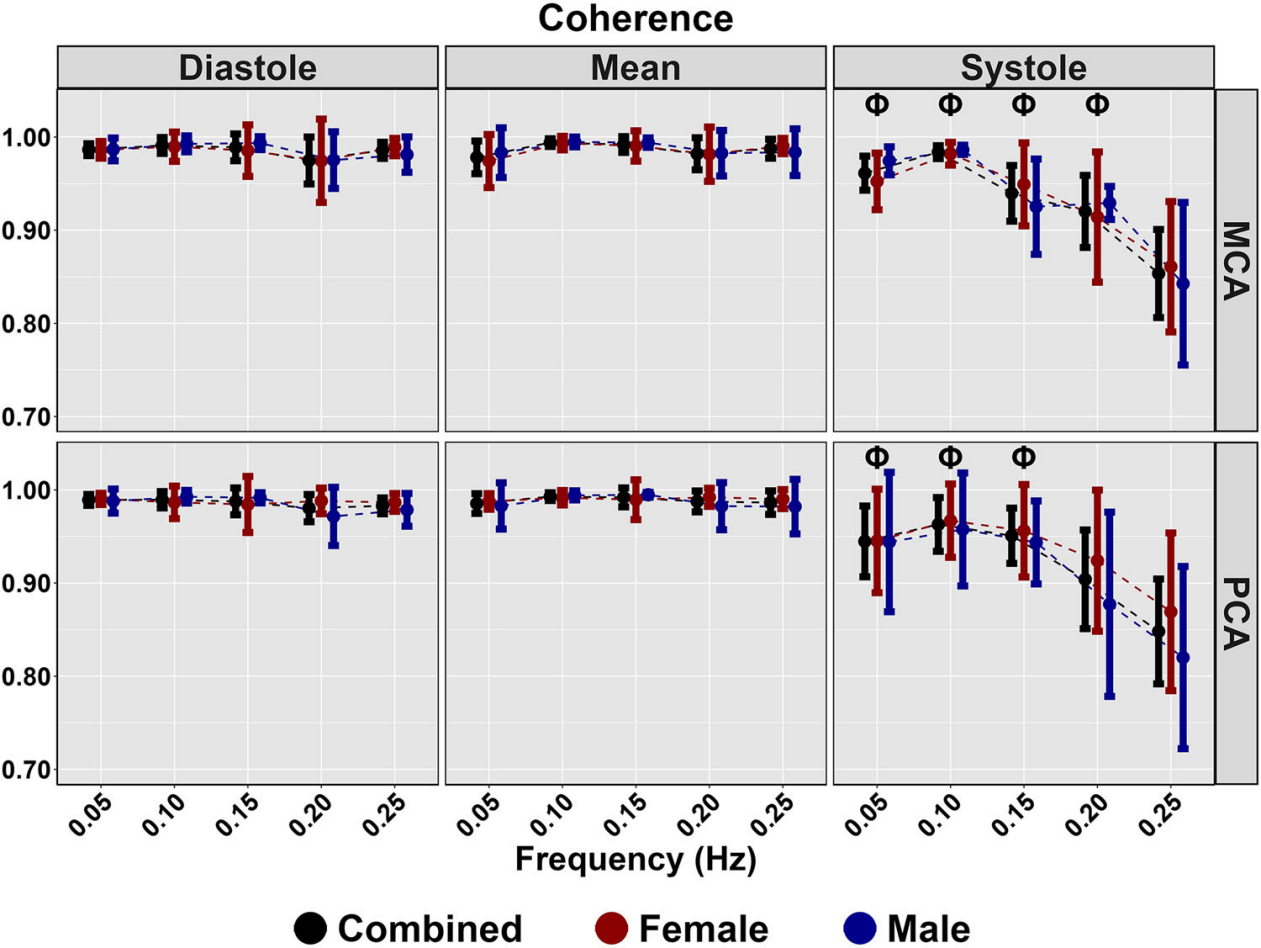
Coherence for MCA and PCA obtained across the cardiac cycle during squat–stand manoeuvres at 0.05, 0.10, 0.15, 0.20 and 0.25 Hz. Data are stratified into females (red; *n* = 9), males (blue; *n* = 7) and combined (black; *n* = 16). The symbol Φ denotes a frequency that differs compared with 0.25 Hz. This was assessed using a linear regression, with frequency (0.25 Hz reference) as the predictor variable and with adjustment for sex (female reference) and order of squat completion. No sex differences were noted. Abbreviations: MCA, middle cerebral artery; PCA, posterior cerebral artery.

**FIGURE 4:**
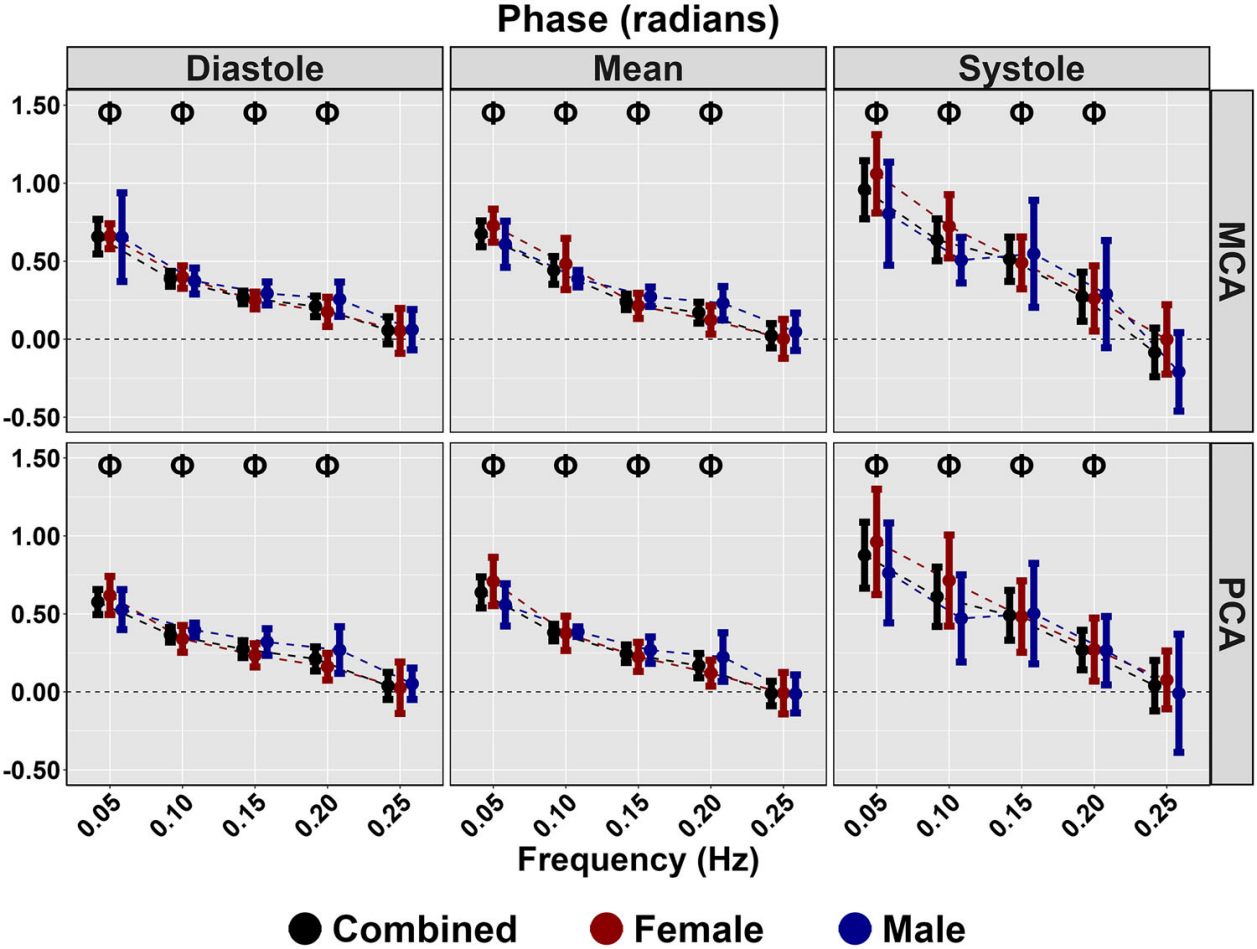
Phase for MCA and PCA obtained across the cardiac cycle during squat–stand manoeuvres at 0.05, 0.10, 0.15, 0.20 and 0.25 Hz. Data are stratified into females (red; *n* = 9), males (blue; *n* = 7) and combined (black; *n* = 16). The symbol Φ denotes a frequency that differs compared with 0.25 Hz. This was assessed using a linear regression, with frequency (0.25 Hz reference) as the predictor variable and with adjustment for sex (female reference) and order of squat completion. No sex differences were noted. Abbreviations: MCA, middle cerebral artery; PCA, posterior cerebral artery.

**FIGURE 5:**
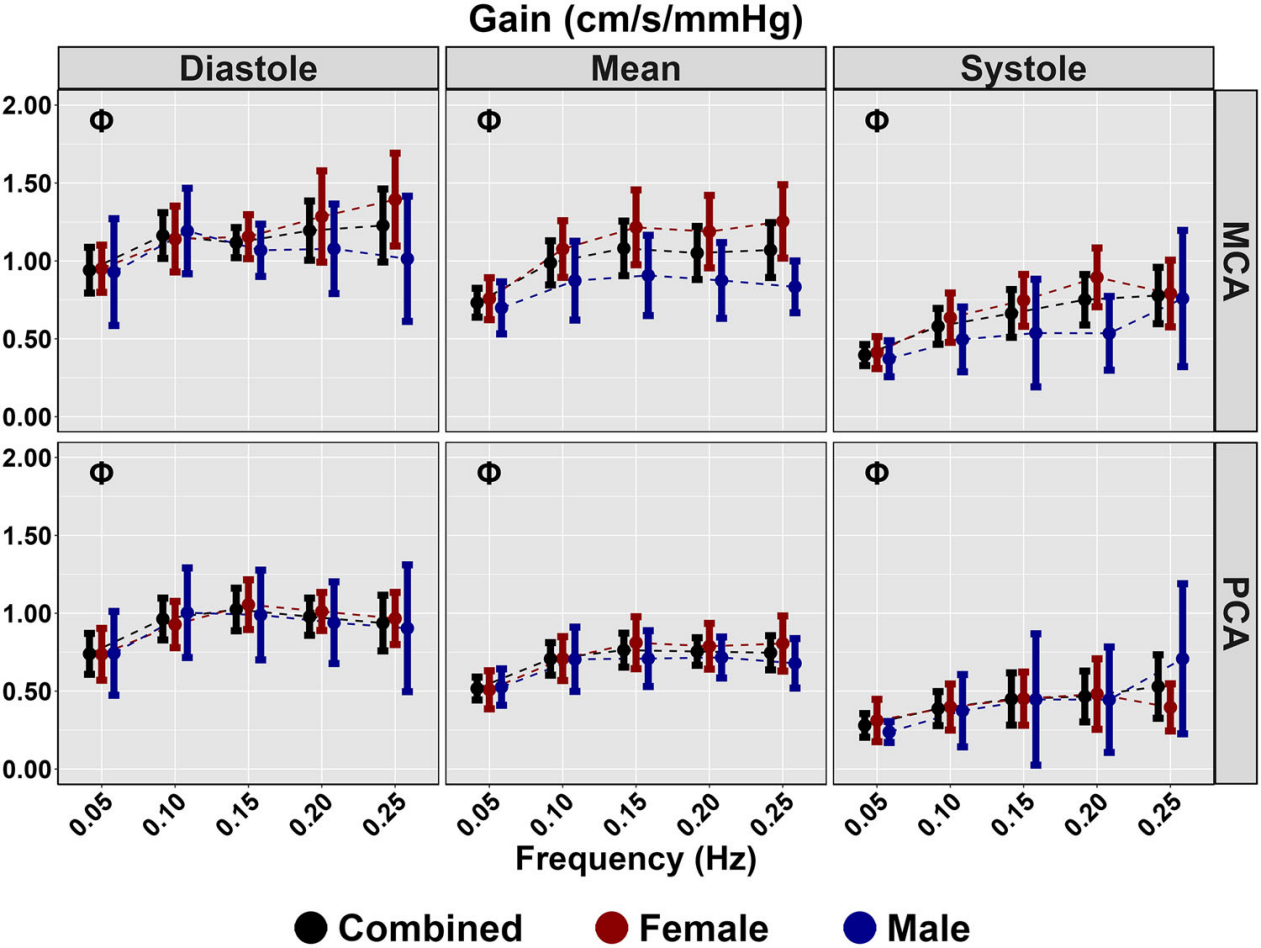
Gain for MCA and PCA obtained across the cardiac cycle during squat–stand manoeuvres at 0.05, 0.10, 0.15, 0.20 and 0.25 Hz. Data are stratified into females (red; *n* = 9), males (blue; *n* = 7) and combined (black; *n* = 16). The symbol Φ denotes a frequency that differs compared with 0.25 Hz. This was assessed using a linear regression, with frequency (0.25 Hz reference) as the predictor variable and with adjustment for sex (female reference) and order of squat completion. Males displayed a lower diastole MCA gain (*P *< 0.001), mean MCA gain (*P *< 0.046) and systole MCA gain (*P* = 0.009). Abbreviations: MCA, middle cerebral artery; PCA, posterior cerebral artery.

**FIGURE 6:**
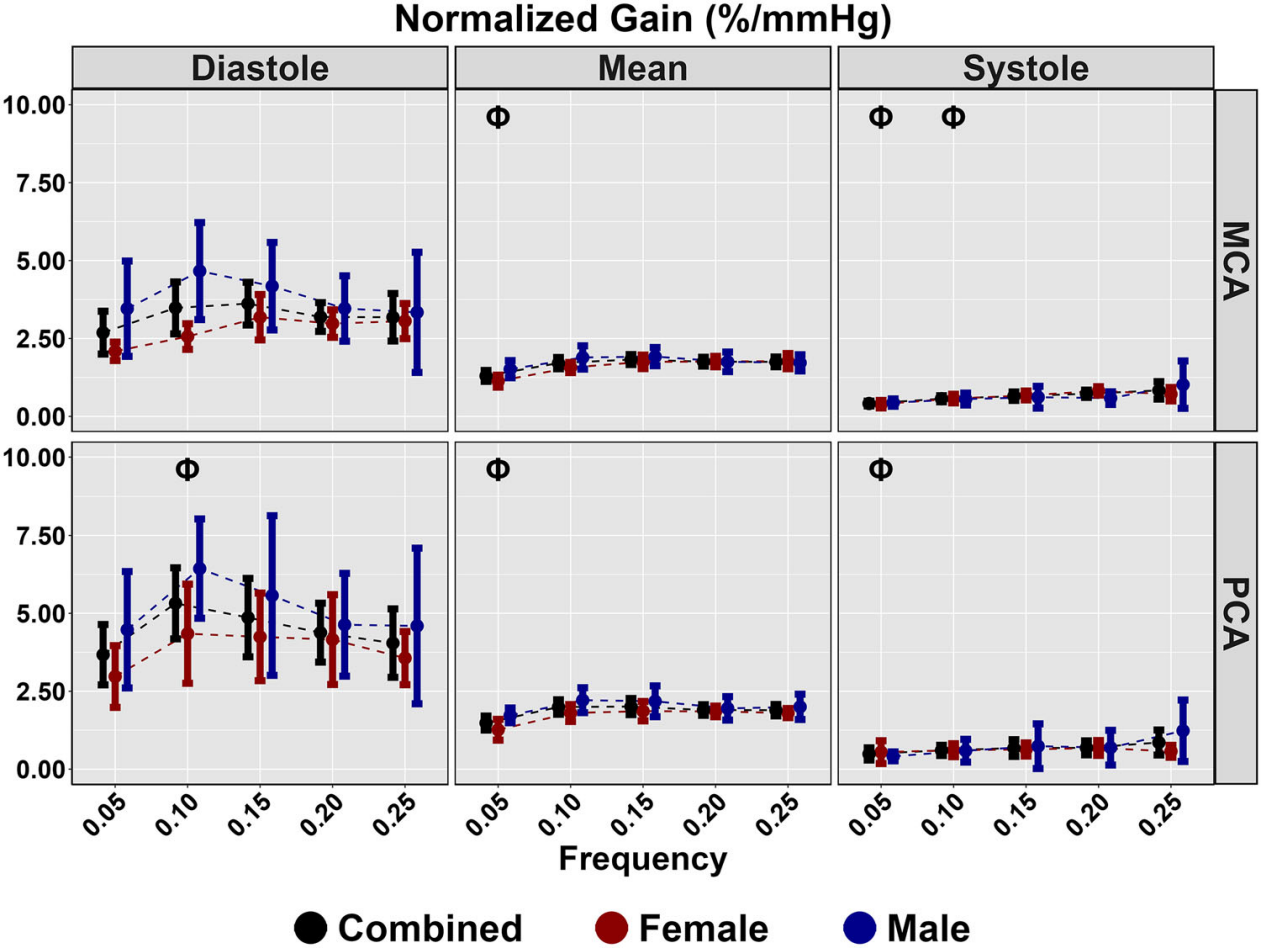
Normalized gain for MCA and PCA obtained across the cardiac cycle during squat–stand manoeuvres at 0.05, 0.10, 0.15, 0.20 and 0.25 Hz. Data are stratified into females (red; *n* = 9), males (blue; *n* = 7) and combined (black; *n* = 16). The symbol Φ denotes a frequency that differs compared with 0.25 Hz. This was assessed using a linear regression, with frequency (0.25 Hz reference) as the predictor variable and with adjustment for sex (female reference) and order of squat completion. Males displayed a greater diastole MCA normalized gain (*P* = 0.019), diastole PCA normalized gain (*P* = 0.006), mean MCA normalized gain (*P *< 0.001) and mean PCA normalized gain (*P* = 0.001). Abbreviations: MCA, middle cerebral artery; PCA, posterior cerebral artery.

**FIGURE 7:**
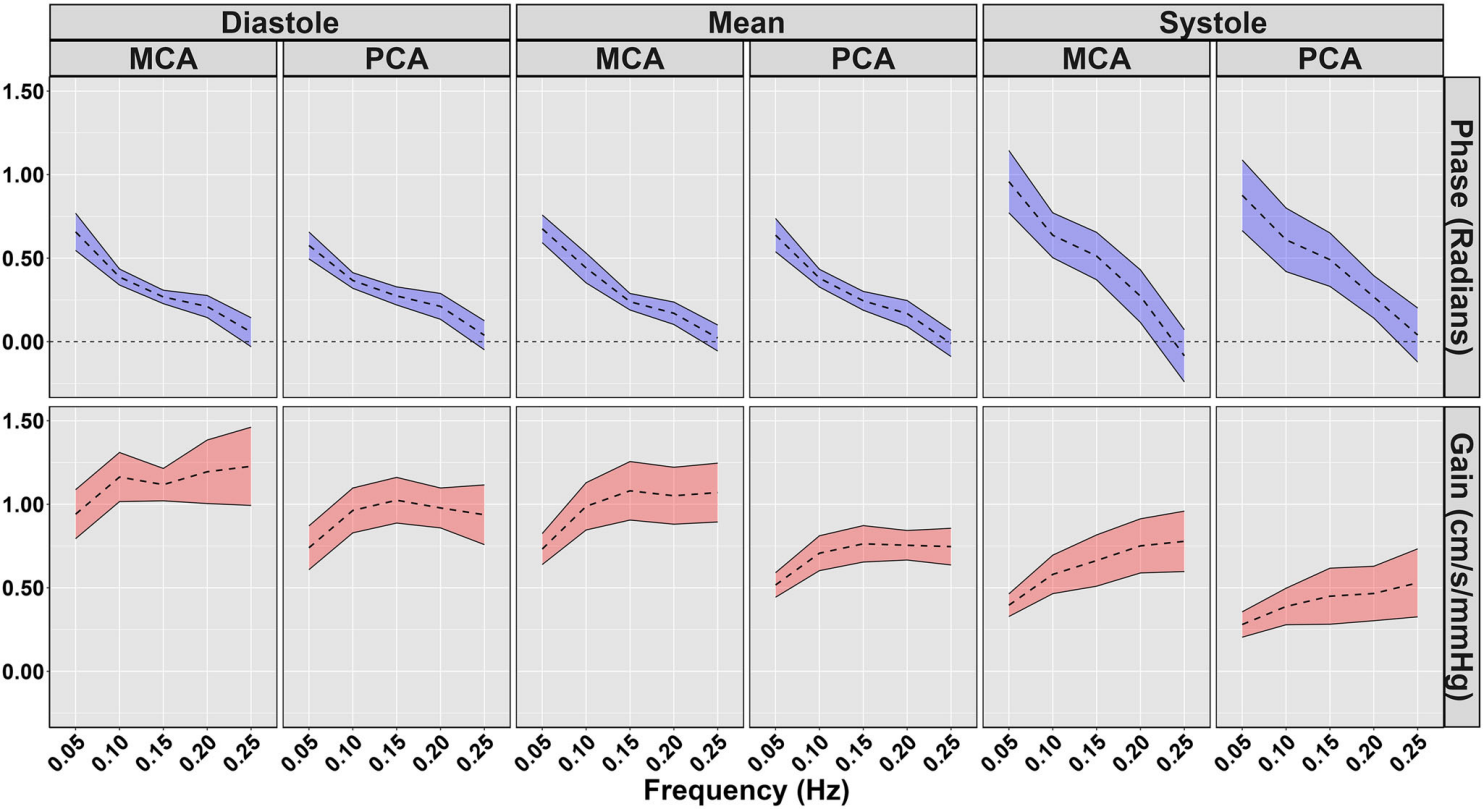
Phase and gain metrics collapsed across participants (*n* = 16; 9 females and 7 males). Data are displayed as the predicted estimates and 95% confidence intervals based on the squat–stand manoeuvre data collected at 0.05, 0.10, 0.15, 0.20 and 0.25 Hz. Abbreviations: MCA, middle cerebral artery; PCA, posterior cerebral artery.

**FIGURE 8:**
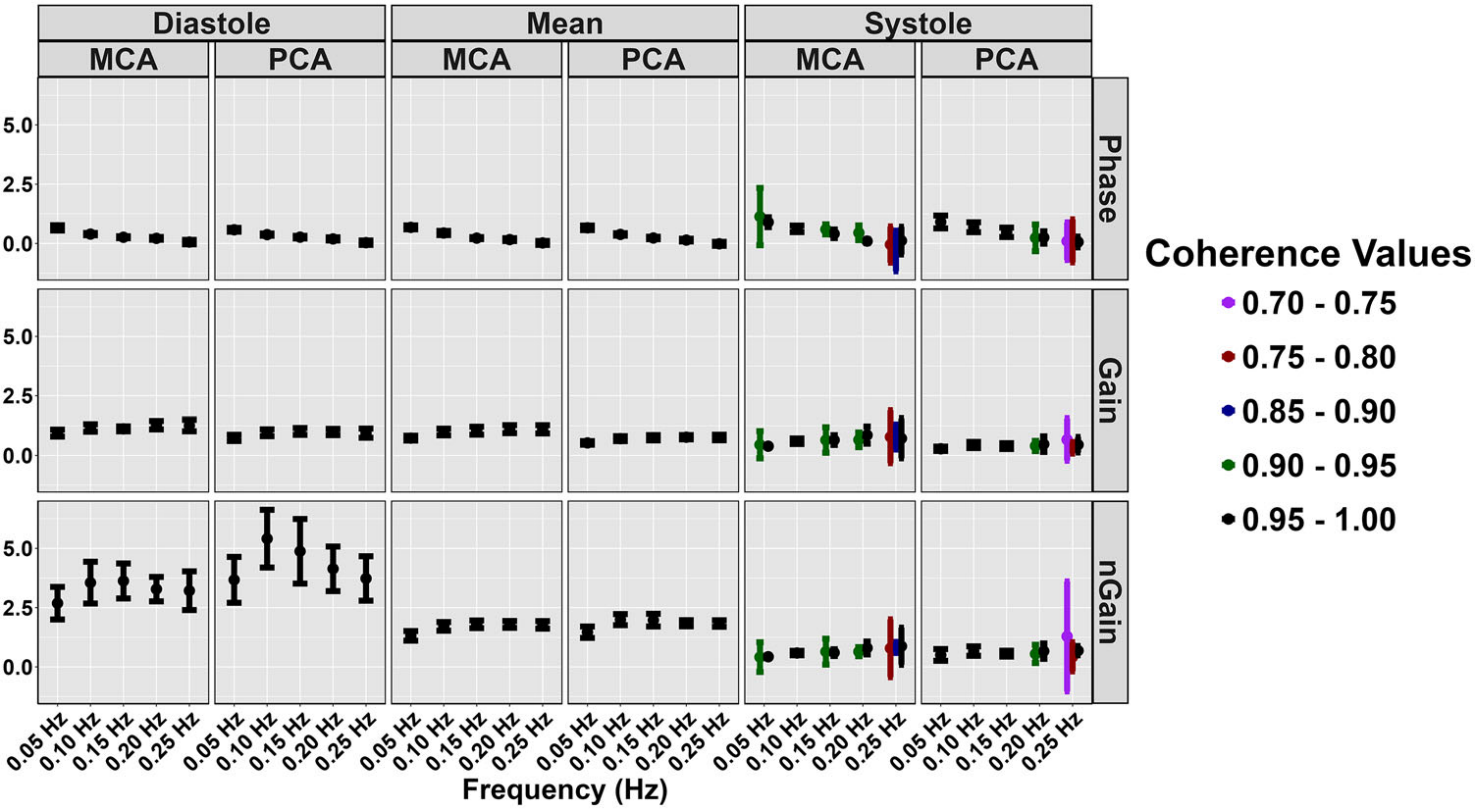
Phase and gain metrics (mean ± 95% confidence intervals) stratified by magnitude of coherence. Data are presented from 0.70 to 1.00 in 0.05 bins for the MCA and PCA during five frequencies of squat–stand manoeuvres. The unequal representation across bins is because 3.3% of the data had a coherence ranging from 0.7 to 0.8, 6.4% of the data had a coherence ranging from 0.8 to 0.9, and 90.3% of the individual data points had a coherence of >0.90. For the data with coherence values ranging from 0.80 to 0.90, 73% of these coherence data occurred at the faster frequencies of 0.15, 0.20 or 0.25 Hz, and 80% also occurred within the systolic aspect of the cardiac cycle. Within the mean aspect of the cardiac cycle, only two values fell below a coherence of 0.90, with one at 0.05 Hz (0.882) and the other at 0.20 Hz (0.881). This provides further validation of the recent the International Cerebrovascular Research Network white paper recommendation #19 stating that repeated squat–stand manoeuvres should be used whenever possible for augmenting coherence during cerebral autoregulation data collections (Panerai et al., 2023). Abbreviations: MCA, middle cerebral artery; PCA, posterior cerebral artery.

Diastolic and mean coherence were similar across all frequencies (all *P >* 0.313), and coherence was greater for all frequencies in systole compared with 0.25 Hz (all *P <* 0.050), aside from 0.20 Hz PCA (*P = *0.218) (Table 3; Figure 3). No sex differences were present for coherence (all *P >* 0.194) (Table 3; Figure 3). Phase was greater at all frequencies compared with 0.25 Hz across the cardiac cycle (all *P <* 0.020); however, no sex differences were present (all *P >* 0.072) (Table 3; Figure 4). Gain was lower for all measures at 0.05 Hz (all *P <* 0.046); however, no differences were present for other metrics at 0.10, 0.15 or 0.20 Hz compared with 0.25 Hz (Table 3; Figure 5). A sex effect was present, with males displaying a lower diastole MCA gain (*P <* 0.001), mean MCA gain (*P <* 0.046) and systole MCA gain (*P = *0.009) (Table 3; Figure 5). Diastole MCAv, mean PCAv, systole MCAv and systole PCAv at 0.05 Hz and diastole PCAv and systole MCAv at 0.10 Hz were lower compared with 0.25 Hz (all *P <* 0.046), with systole MCA gain at 0.10 Hz additionally being lower (*P = *0.013) (Table 3; Figure 6). However, no other differences were noted in nGain (all *P >* 0.080) (Table 3; Figure 6). Males displayed a greater diastole MCA normalized gain (*P = *0.019), diastole PCA normalized gain (*P = *0.006), mean MCA normalized gain (*P <* 0.001) and mean PCA normalized gain (*P = *0.001) (Table 3; Figure 6). Figure 7 displays the predictive phase and gain values ranging from 0.05 to 0.25 Hz with CIs displaying the approximate value where phase reaches zero and where gain begins to plateau. Finally, Figure 8 displays phase, gain and nGain data stratified into coherence groupings of 0.05 Hz, where a lower coherence was associated with slightly more variation. However, it should be noted, that these were produced from three or four participants, thus leading to inflated CIs.

## 4 DISCUSSION

The present investigation built upon the ‘considerable interest’ notion presented by Panerai et al. (2019) and used the technique of repeated SSMs to examine the upper frequency limit cut‐off point for CA (Panerai et al., 2019). This technique is capable of eliciting the greatest physiological signal‐to‐noise ratio and thus providing the most reliable estimates of TFA parameters (Smirl et al., 2015). Counter to our hypotheses expecting consistent agreement with TFA phase and gain, it was revealed that there are different indexes for the upper frequency limit of CA based upon the TFA metric that was examined. The upper frequency limit of CA based upon TFA phase is within the range of 0.20–0.25 Hz (Table 3; Figure 4), which is in agreement with the theoretical upper limit from the Gaussian distribution (0.24 Hz) as calculated with TFA phase by Panerai et al. (2019). In contrast, the upper frequency limit of CA indexed with TFA gain is within the range of 0.05–0.10 Hz (Table 3; Figure 5). This is in agreement with the PPR model (which works on the changes in amplitude of CBv for a given change in blood pressure, similar to the TFA gain metric) (Taylor et al., 2014). However, as previously stated, this approach produces a robust autoregulatory plateau in only ∼25% of individuals (Burma, Griffiths, Smirl, et al., 2024; Saleem et al., 2015). Nevertheless, the present data set appears to validate both claims made within the literature for the upper frequency limit of CA (Panerai et al., 2019; Taylor et al., 2014). The present data set also built upon the prior work in the CA field, by stratifying the effect of CA changes across the cardiac cycle and by adjusting for biological sex on these outcome measures. No sex differences were found when examining TFA phase. However, absolute MCA TFA gain across the cardiac cycle was blunted in males (compared with females), and TFA nGain was elevated in males (compared with females) for diastole and mean values in both the MCA and PCA.

The present findings between the TFA phase and gain metrics appear to have conflicting interpretations, with TFA phase indicating a much higher upper limit, between 0.20 and 0.25 Hz (Table 3; Figure 4), whereas TFA gain presents a much lower limit, between 0.05 and 0.10 Hz (Table 3; Figures 5 and 6). When comparing the present findings with those in the broader literature (Panerai et al., 2019; Taylor et al., 2014; Tzeng et al., 2012), there are some striking consistencies within the individual CA metrics. The present findings for TFA phase are in agreement with the data presented by Tzeng et al. (2012), which show an inherent decrease in TFA phase beginning at ∼0.04–0.07 Hz and reaches a plateau of 0 radians at ∼0.20 Hz across a wide range of $P_{\mathrm{ET},CO_{2}}$ levels (from +15 to −20 mmHg around eucapnia). Although these prior data were collected from spontaneous oscillations in blood pressure and only for the mean aspect of the cardiac cycle in the MCA, this decrease for TFA phase up to 0.20 Hz is highly consistent with the present findings (Figure 4) and those predicted by Panerai et al. (2019). However, the present findings were able to extend the prior observations for TFA phase and confirm that the upper frequency limit for this CA metric is consistent across all aspects of the cardiac cycle and in female and male biological sexes.

With respect to the relatively low frequency of 0.05–0.10 Hz for the upper frequency limit of CA observed for both absolute and normalized TFA gain metrics in the present investigation (Table 3; Figures 5 and 6), this finding is again largely consistent with the prior literature, even with measures being performed across multiple CA assessment techniques (Taylor et al., 2014; Tzeng et al., 2012). The absolute TFA gain data from the study by Tzeng et al. (2012) are similar to the present findings (Figure 5) in that they both revealed plateaus beginning to occur at ∼0.10 Hz. The prior data also revealed that the plateau in TFA gain for the mean MCA response extended from +15 mmHg hypercapnia to −10 mmHg hypocapnia, which is harmonious with the eucapnia findings in the present study. Under the more extreme level of hypocapnia of −20 mmHg, the TFA gain plateau was extended to ∼0.15 Hz, which is consistent with the hypocapnia data presented by Panerai et al. (2019). The nGain findings (Figure 6) in the present report, however, differ slightly from those reported by Tzeng et al. (2012), in that the prior investigation showed a TFA nGain plateau occurring at a slightly higher frequency (∼0.15 Hz), whereas the present data revealed the plateau occurring between 0.05 and 0.10 Hz. It is possible that the way in which the data were obtained might have played a role in this slight discrepancy for this CA metric, because the prior work was performed during spontaneous blood pressure oscillations, whereas the present data were collected with blood oscillations driven via repeated SSMs (Smirl et al., 2015). Repeated SSMs have been shown consistently to augment the signal‐to‐noise ratio between blood pressure and CBv (Barnes et al., 2018; Batterham et al., 2020; Birch et al., 1995; Burma, Copeland, Macaulay, et al., 2020; Claassen et al., 2009; Newel et al., 2022; Smirl et al., 2015), which enhances the reliability and interpretability of the associated TFA phase and gain metrics (Smirl et al., 2015).

Comparing the present findings from the TFA‐based analysis of CA with the prior work using PPR (Taylor et al., 2014) is not as simple. PPR is a non‐parametric regression model that functions to compare ‘ridge functions’ of linear combinations of predictor variables (Friedman & Stuetzle, 1981). In terms of using PPR analysis in CA, there is often only one ridge function used (linear regression between blood pressure and CBv), which is indexed for a falling slope, autoregulatory plateau and a rising slope, with the slope changes being indicative of autoregulatory gain (Hamner & Tan, 2014; Tan, 2012; Tan et al., 2013; Taylor et al., 2014). Through this analytical approach with driven oscillations in blood pressure via oscillatory lower‐body negative pressure (−30 mmHg), it was revealed that oscillations occurring at >0.07 Hz were largely linear in nature, with changes in blood pressure largely being passed on directly to the CBv and with virtually no dampening occurring (Taylor et al., 2014). Given that PPR quantifies the change in millimetres of mercury for blood pressure versus the change in centimetres per second for CBv at a given frequency of interest, this metric is most comparable to the absolute TFA gain index in the present results. Examining the findings within the present investigation (Figure 5) in comparison to the prior work by Taylor et al. (2014), there are consistencies in ascertaining the upper frequency limit of CA for TFA gain to occur within the range of 0.05–0.10 Hz. However, prior work by other authors applying PPR for assessing CA has suggested that this approach should be used with caution, because the expected sequence of a falling slope, autoregulatory plateau and a rising slope was present in only 28% (Saleem et al., 2015) or 23% (Burma, Griffiths, Smirl, et al., 2024) of the data.

Furthermore, the present investigation was able to extend the understanding surrounding the upper frequency limit of CA associated with TFA gain beyond the typical mean CBv data. The present data (Table 3) revealed there are some subtle differences between biological sexes, with absolute TFA gain shown to be slightly blunted in males (compared with females) for diastole, mean and systole, whereas TFA nGain was augmented in males (compared with females) for diastole and mean values. This is an important finding, because it confirmed prior work in the CA field revealing that females had lower TFA nGain for mean CBv than their male counterparts (Favre & Serrador, 2019) and extended this finding to diastole, in addition to showing the opposite results for the absolute TFA gain results for the mean and systolic aspects of the cardiac cycle (Figures 5 and 6). The different outcomes with respect to biological sex differences in absolute versus normalized TFA gain across the cardiac cycle are likely to be associated with the mathematical construct of these metrics (Tzeng et al., 2012). However, despite the sex differences in TFA metrics, Figures 4, 5, 6, 7 demonstrate similar trend lines, highlighting that the absolute differences associated with biological sex might not reflect any differences in the upper frequency limit of autoregulation. Furthermore, Figure 7 highlights similar findings for cardiac cycle phase and vessel of insonation. Although the phase and/or gain 95% CIs do not overlap at certain frequencies, these appear to plateau or reach zero at a similar frequency (Figure 7).

Additionally, Table 1 denotes that the 95% CIs did not overlap for females and males in MCAv, but did for PCAv, explaining the augmented gain findings within females in the MCAv (Table 3; Figure 5). This is consistent with our prior research investigations, which have shown that females generally have higher CBv in comparison to males (Table 1) (Burma, Wassmuth, Kennedy, et al., 2023, 2021; Johnson et al., 2023). The higher CBv in females is attributed primarily to differences in vascular anatomy, particularly the smaller cross‐sectional area of the cerebral arteries and slightly lower haematocrit levels (reduced blood viscosity). This physiological characteristic can be explained by the principles of fluid dynamics; for a given volume of blood, flow velocity must increase as the cross‐sectional area of a vessel decreases. Despite the smaller cross‐sectional area, females displayed a higher gain, which appears to be paradoxical of known MCAv and PCAv differences in gain (Burma, Copeland, Macaulay, et al., 2020; Reehal et al., 2021; Smirl, Tzeng, Monteleone, et al., 2014). It has been proposed that a larger diameter leads to a greater gain (Burma, Copeland, Macaulay, et al., 2020; Reehal et al., 2021; Smirl, Tzeng, Monteleone, et al., 2014); however, in theory this would lead to males having an augmented TFA gain compared with females. Therefore, these differences in gain are likely to be associated with the nature of the fast Fourier transform, whereby a larger velocity will produce a greater CBv power spectral density and thus a greater gain for a similar change in blood pressure (Claassen et al., 2016; Panerai et al., 2023). Nonetheless, the lack of observable differences in the upper frequency limit across the cardiac cycle, between sexes and between vessels is likely to mean that the difference in cerebral conduit vessel cross‐sectional area between sexes has nominal influence of the upper limitation of CA.

As discussed by Panerai et al. (2019), the physiological underpinnings of the upper frequency limit are likely to be attributable to the capabilities of the vascular smooth muscle. The present investigation was completed in eucapnic conditions, whereas the previous report completed additional assessments during hypocapnia and hypercapnia (Panerai et al., 2019). Although blood pressure and heart rate were maintained during the protocols, the upper frequency limit was influenced by changes in the arterial partial pressure of carbon dioxide (Panerai et al., 2019). This is consistent with seminal work by Birch et al. (1995), who completed SSMs during breathing challenges, noting lower phase (i.e., lower autoregulation) during hypercapnia and higher phase during hypocapnia (i.e., greater autoregulation). Panerai et al. (2019) theorized that this is likely to be attributable to alterations in pH altering endothelial function and calcium influx, rather than a vascular smooth muscle shortening hypothesis (i.e., greater time to shorten vascular smooth muscle during hypercapnia) (Capellini et al., 2013; Nazarov et al., 2000). Finally, it is important to consider that respiratory frequency could influence metrics derived at the upper frequency limit, because 0.25 Hz corresponds to respiratory rate of 15 breaths/min. This would have nominal influence on TFA metrics deduced using SSMs, because the power spectral estimates greatly override other physiological signals (Smirl et al., 2015). Nevertheless, this would impact TFA metrics derived from spontaneous values, if the respiratory rate fell into the frequency range of autoregulation. However, the effects of respiratory rate would be minimal in comparison to changes in arterial carbon dioxide concentrations (Panerai et al., 2019).

The final question to be addressed based on the present findings is: which upper frequency limit accurately represents the overall CA regulatory mechanism? Unfortunately, there is no simple answer to this question, because it all depends on the angle from which one examines the data. Do we examine the changes with respect to the difference in timing between blood pressure and CBv (TFA phase) or do we look at the change in volume of blood being passed through the cerebrovasculature (TFA gain)? For examining the TFA phase side of the coin, it has been proposed that this could potentially be the most appropriate method. The phase lead for CBv changes preceding those of systemic blood pressure could be a result of the mathematical construct resulting from how the cerebrovascular resistance index (CVRi) is calculated (CVRi = blood pressure/CBv) (Hughson et al., 2001). However, there is a conflicting perspective for the notion of phase lead, and that it is not a mathematical construct, but occurs as a result of autonomic (probably sympathetic) neural control within the cerebrovasculature that enables the brain to modulate and control its blood flow from the microvasculature upstream to the conduit vessels to control blood flow across the brain (Cencetti et al., 1999). From this perspective, the dilatation or constriction within the cerebral blood vessels plays an extremely crucial role in the notion of phase lead, and the only notion for an upper frequency limit for CA would be the point at which CVRi becomes constant and occurs when the phase reaches 0 radians. In contrast, it could also be discussed that at the point at which the optimal volume of blood is being transmitted to the brain (TFA gain), there are intrinsic factors at play with respect to the Monro–Kellie doctrine (Abercrombie, 1836) that limit the volume of substances within the cranium in order to maintain/optimize intracranial pressures (Brassard et al., 2021). From this perspective, the point at which TFA gain plateaus could be considered as the most important indicator of the true upper frequency limit of CA. This is because it would indicate that the CVRi is at the point where it is most actively regulating against further increases in the volume of blood entering the cranium and limiting increases to intracranial pressure. Unfortunately, the present data set is unable to disentangle which of these two sides of the coin is ultimately correct, because it appears as though TFA phase and gain (which are both valid aspects of CA) are not able to be used interchangeably owing to the inherent quandary associated with quantifying CA (Tzeng et al., 2012). In the report by Tzeng et al. (2012), it was shown that phase and gain often have very limited correlations with one another, despite both being representative aspects of CA. As such, it is recommended that researchers report both TFA phase and gain values in their research investigations, in alignment with the CARNet white papers (Claassen et al., 2016; Panerai et al., 2023), because it appears that they both capture important and independent information with respect to the ‘black‐box’ nature of CA (Willie et al., 2014).

Finally, as the present data highlight, there are concerns within the broader CA field with respect to the highly variable quality of some studies within the CA literature that can confound the interpretations being made across studies using various methodological approaches and populations. For more in‐depth information on these domains, it is recommended that readers examine the works by Angarita‐Jaimes et al. (2014), Brassard et al. (2023), Kostoglou et al. (2024), Panerai (1998), Simpson et al. (2022) and Tzeng et al. (2012). It has also been suggested that the conundrum of approaches for assessing CA in both the frequency domain (Claassen et al., 2016; Panerai et al., 2023) and the time domain (Kostoglou et al., 2024) can result in difficulties for investigators to know which approaches are comparable to which others and how best we should approach the dissemination of this knowledge as a collective CA field. The present study highlights this point, because it is shown clearly by our results that TFA phase and TFA gain measures do not reflect the same aspects of CA and, as pointed out previously, appear to contradict each other for the upper frequency limit of dynamic CA (Figure 7). This leads to the question of whether it is best to omit certain aspects of data from studies to streamline the interpretation of the findings, because the apparent conflicting nature of the TFA phase and gain metrics is potentially adding to the problem of divergent findings in the CA field (Tzeng et al., 2012), which could even be interpreted by some individuals as these findings adding to the inconsistency and poor science often found in the CA literature. To individuals with this belief, we can only state that it is best for all researchers to follow the best‐practice standards for their respective fields and abide by the white paper guidelines wherever they are made available. In the case of the CA field, the CARNet group has published both frequency‐domain (Panerai et al., 2023) and time‐domain (Kostoglou et al., 2024) versions. The present data were collected in full agreement with all aspects of these white papers regarding data collection, data processing and data analysis recommendations from this world‐leading group, which should diminish any concerns that this study has added to the perceived problem of inconsistency and potentially poor science being performed within the CA field. Instead, it appears as though the differential findings in the present data (Figure 7) clearly highlight that there is a need for researchers to make direct comparisons whenever possible when results are compared across investigations. It is best to make as many ‘apples with apples’ comparisons as possible. In fact, it is recommended that interpretations within the CA field are not made broadly, such as simply stating that CA is impaired or improved, but instead should highlight the specific aspect of CA that is being compared across studies (and ideally have the data collected in the same manner, driven versus spontaneous blood pressure oscillations) and should be processed in alignment with the CARNet white papers. Without this level of scientific rigour being followed by the field, it is possible to be part of the problem, add to the inconsistency and lead to poor science being found in the CA literature.

### 4.1 Limitations

Transcranial Doppler ultrasound has an inherent limitation as a result of its ability only to capture data related to the velocity of red blood cells within the main conduit arteries of the brain, instead of assessing cerebral blood flow directly. Publications that have examined high‐resolution magnetic resonance imaging for examining the cerebrovasculature across various levels of $P_{\mathrm{ET},CO_{2}}$ have shown that when $P_{\mathrm{ET},CO_{2}}$ levels are within 8 mmHg of eucapnia, vessel diameter is relatively consistent (Ainslie & Hoiland, 2014; Coverdale et al., 2014; Verbree et al., 2014). Given that data collection was performed at eucapnic levels for the present investigation, CBv data are likely to be a good surrogate for cerebral blood flow. The exact frequency point of the upper limit of CA was not determined precisely to the 0.01 Hz accuracy level within the present study. This is because the repeated SSMs were performed at five frequencies across the points between 0.05 and 0.25 Hz in order to limit the physical burden on participants and enable the data collections to occur within a reasonable time frame. Future research could be performed to examine the upper limit associated with TFA gain by performing manoeuvres at 0.05, 0.06, 0.07, 0.08, 0.09 and 0.10 Hz. Likewise, another future research investigation could be performed to examine TFA phase by performing manoeuvres at 0.20, 0.21, 0.22, 0.23, 0.24 and 0.25 Hz to enhance the accuracy associated with the upper frequency limit for this aspect of CA.

The participants within the present study were mainly recreationally active, healthy university undergraduate and graduate students; therefore, the results might not be transferable directly to elderly, clinical or highly trained populations (Ainslie et al., 2008; Claassen et al., 2009; Labrecque et al., 2017; Patricios et al., 2023). Reduced CA is generally a pathophysiological consequence of disease progression; therefore, it is likely that the upper frequency limit would be lower in clinical populations (Claassen et al., 2021). Nevertheless, further work is warranted to confirm this proposition. Previous work has demonstrated that cardiorespiratory fitness influences CA estimates (Labrecque et al., 2017); however, no notable sex differences in physical activity levels were present. Additionally, although sex was adjusted in the regression models, specific sex differences in the upper frequency limit were not delineated owing to the small sample size. Furthermore, differences in cardiac cycle measures were not compared directly, whereby the results were stratified into the different components, with Figure 7 denoting similar trends. Nonetheless, Figures 4 and 5 demonstrate that both sexes and cardiac cycles displayed similar decreases in phase from 0.05 to 0.25 Hz, with a plateau occurring at ∼0.10 Hz in gain. The phase of the menstrual cycle was not controlled for in the present investigation. Prior CBv research has shown that there is minimal change to CA metrics across the menstrual cycle (Favre & Serrador, 2019), hence the present findings are likely to be generalizable to testing females at any point during the early follicular, late follicular and luteal phases of the menstrual cycle. Furthermore, contraceptive usage was not determined in the present investigation, which warrants further investigation to understand how this influences CA. Finally, the directional sensitivity of the cerebral pressure–flow response was not examined within the present investigation. Prior research has shown that there are frequency‐dependent effects associated with cerebral hysteresis within driven oscillations; therefore, future research is warranted to ascertain whether there is an upper limit associated with this aspect of CA (Labrecque et al., 2022; Panerai et al., 2018).

## 5 CONCLUSION

The present results confirm that there are different interpretations available for the upper frequency limit associated with CA, which is consistent with the broader literature. When examining CA with respect to the amplitude changes in blood pressure that are transferred to CBv (TFA gain), it appears as though the upper frequency limit for CA is between 0.05 and 0.10 Hz, confirming the prior findings with PPR approaches. However, when the upper frequency limit for CA is assessed in relationship to the timing buffer between blood pressure and CBv (TFA phase) the limit occurs at a higher frequency and is between 0.20 and 0.25 Hz. Finally, the present results confirm the prior work showing that the TFA phase is consistent across biological sexes and extend this notion for the various aspects of the cardiac cycle, whereas TFA gain has some subtle biological sex differences present when the cardiac cycle is taken into consideration. This enhanced understanding with respect to the upper frequency limit of CA is an important consideration for impairments in cerebrovascular function associated with clinical conditions, changes in cardiorespiratory fitness and the ageing process.

## AUTHOR CONTRIBUTIONS

Joel S. Burma, James K. Griffiths and Jonathan D. Smirl conceived and designed the study. Joel S. Burma, Matthew G. Neill, Elizabeth K. S. Fletcher, Brooke E. Dennett, Nathan E. Johnson and Raelyn Javra performed the experiments. Joel S. Burma performed data analysis. Joel S. Burma and Jonathan D. Smirl wrote the manuscript. Matthew G. Neill, Elizabeth K. S. Fletcher, Brooke E. Dennett, Nathan E. Johnson, Raelyn Javra and James K. Griffiths contributed to revising the manuscript. All authors have approved the final version of the manuscript and agree to be accountable for all aspects of the work in ensuring that questions related to the accuracy or integrity of any part of the work are appropriately investigated and resolved. All persons designated as authors qualify for authorship, and all those who qualify for authorship are listed.

## CONFLICT OF INTEREST

The authors declare no conflicts of interest.

## Supporting information

Figure S1. Transfer Function Analysis Coherance estimates produced during 5 different squat‐stand manoeuvre frequencies stratified by sex.

Figure S2. Transfer Function Analysis Pas estimates produced using 5 different squat‐stand manoeuvre frequencies stratified by sex.

Figure S3. Transfer Function Analysis Gain estimates produced using 5 different squat‐stand manoeuvre frequencies stratified by sex.

Figure S4. Transfer Function Analysis Normalized Gain estimated produced using 5 different squat‐stand manoeuvre frequencies stratified by sex.

## Data Availability

Data accompanying this manuscript are available upon reasonable request to the corresponding author (Joel S. Burma).
