# Supplementary figures and images for: Examining the upper frequency limit of dynamic cerebral autoregulation: Considerations across the cardiac cycle during eucapnia
Source: Exp Physiol. 2024 Oct 9;109(12):2100–21. doi: 10.1113/EP091719 (PMC11607623; doi:10.1113/EP091719)

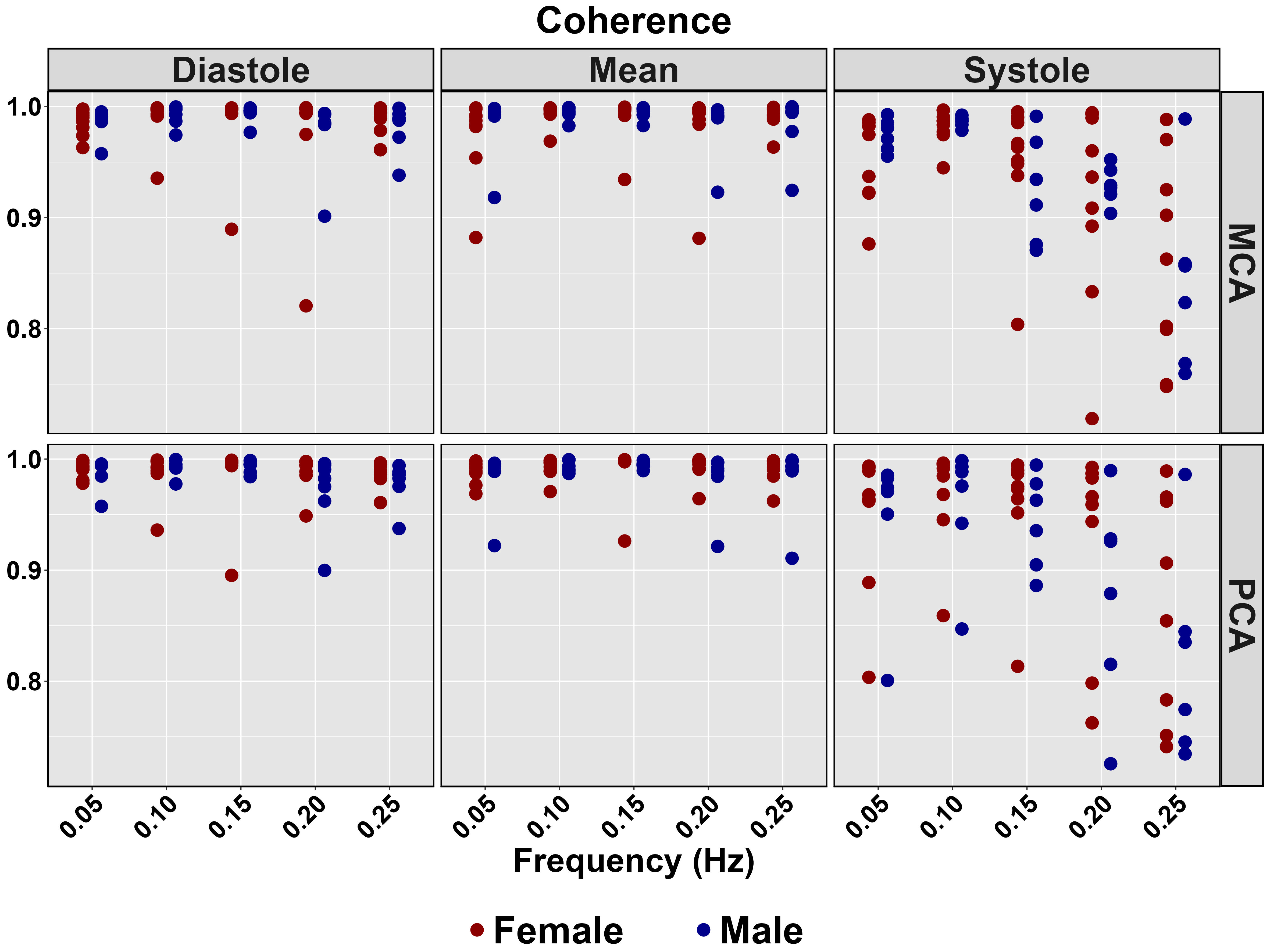

Supplement: Supplementary file 1 — Figure S1. Transfer Function Analysis Coherance estimates produced during 5 different squat‐stand manoeuvre frequencies stratified by sex. [file EPH-109-2100-s002.jpg]

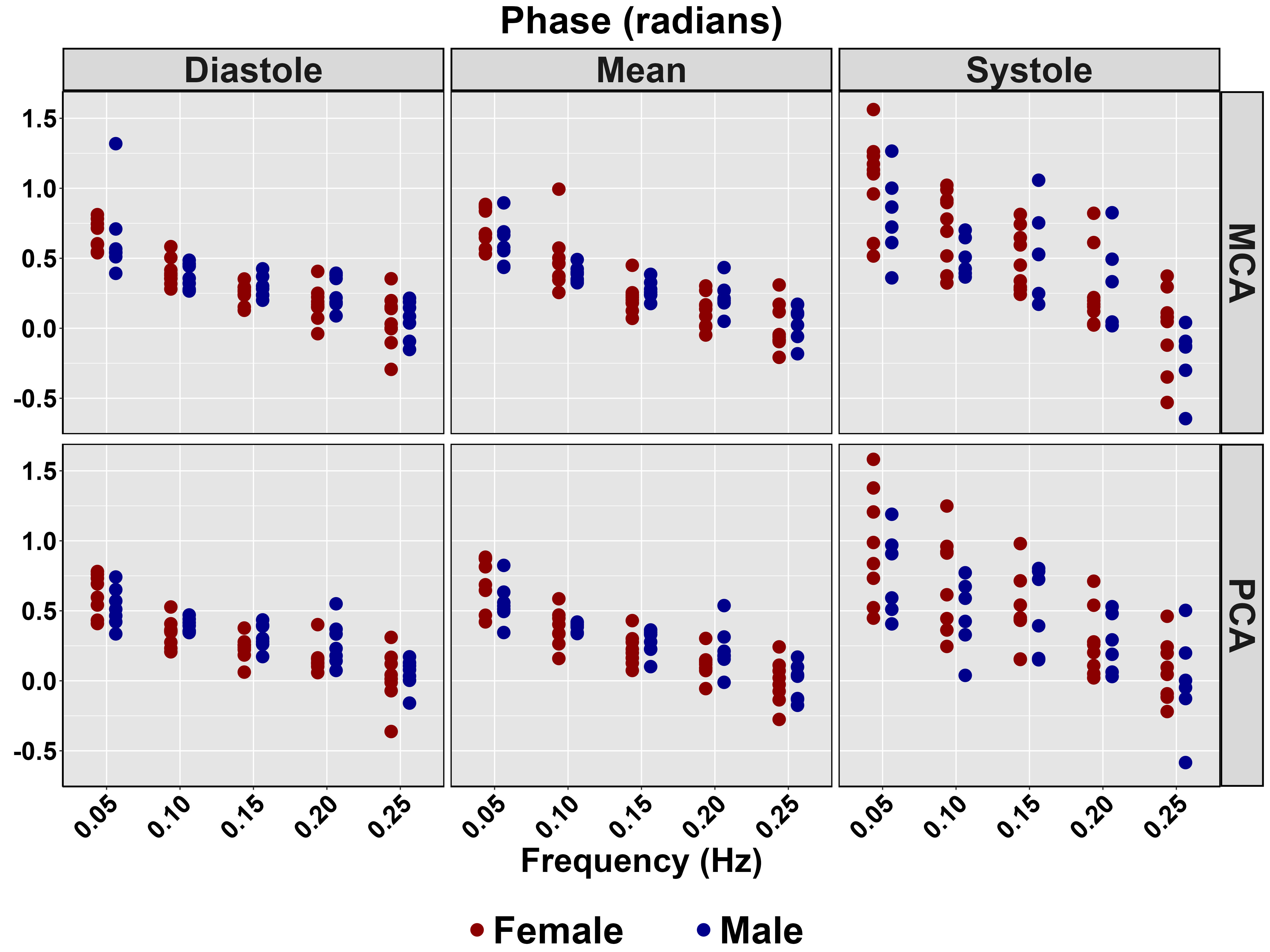

Supplement: Supplementary file 2 — Figure S2. Transfer Function Analysis Pas estimates produced using 5 different squat‐stand manoeuvre frequencies stratified by sex. [file EPH-109-2100-s003.jpg]

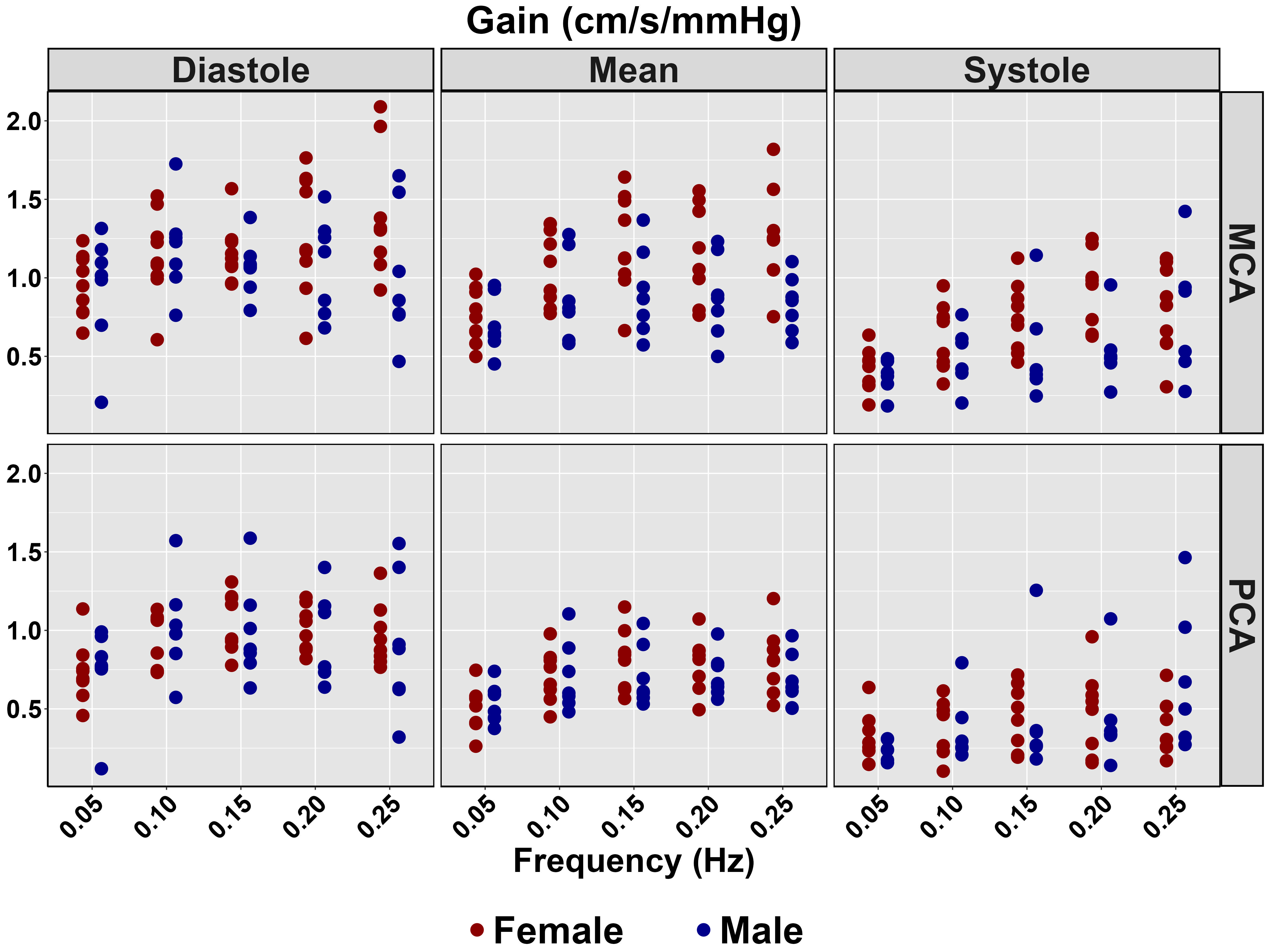

Supplement: Supplementary file 3 — Figure S3. Transfer Function Analysis Gain estimates produced using 5 different squat‐stand manoeuvre frequencies stratified by sex. [file EPH-109-2100-s001.jpg]

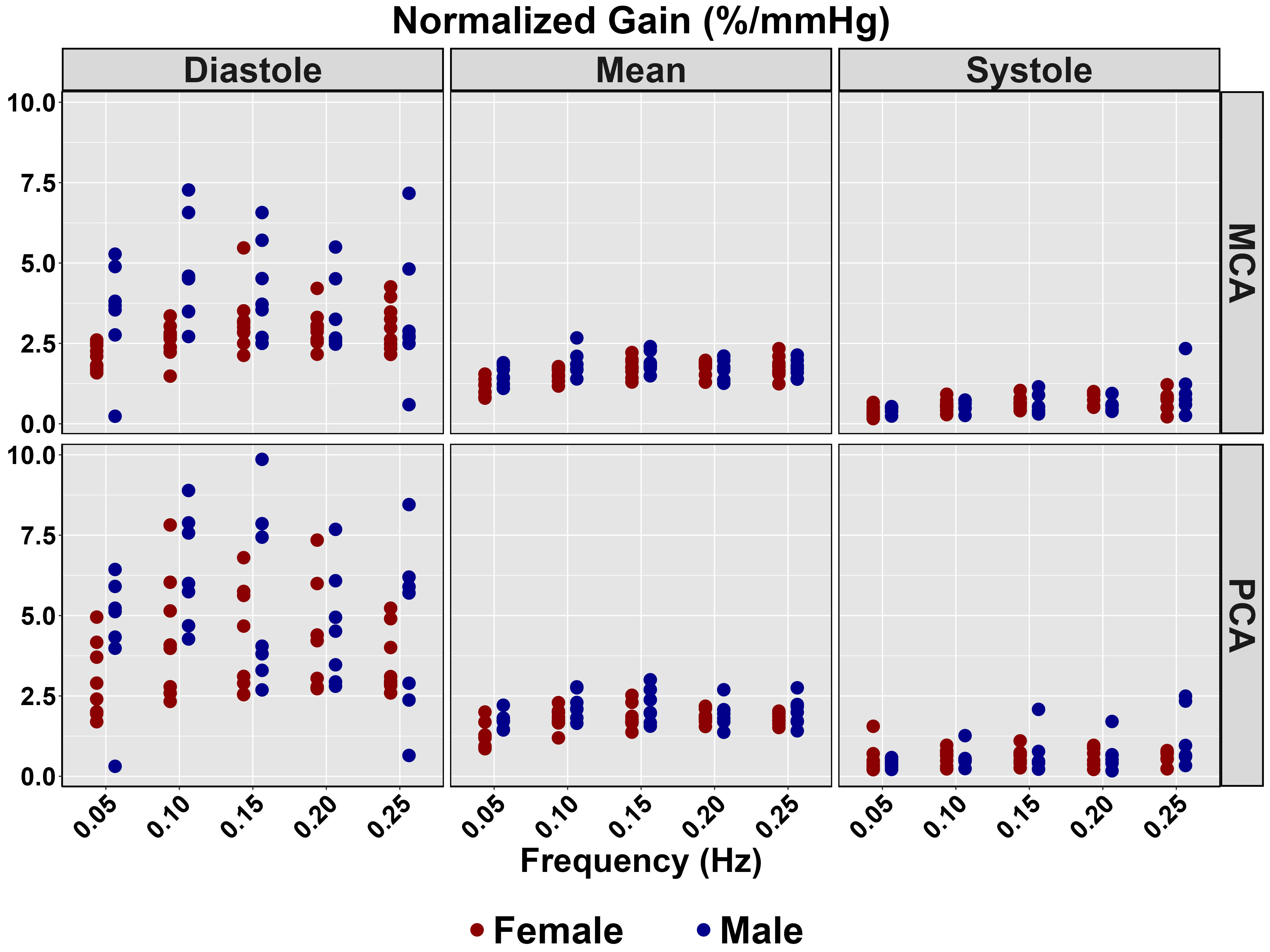

Supplement: Supplementary file 4 — Figure S4. Transfer Function Analysis Normalized Gain estimated produced using 5 different squat‐stand manoeuvre frequencies stratified by sex. [file EPH-109-2100-s004.jpg]
